# MiMIR: R-shiny application to infer risk factors and endpoints from Nightingale Health’s ^1^H-NMR metabolomics data

**DOI:** 10.1093/bioinformatics/btac388

**Published:** 2022-06-13

**Authors:** D Bizzarri, M J T Reinders, M Beekman, P E Slagboom, E B van den Akker

**Affiliations:** Molecular Epidemiology, Biomedical Data Sciences, LUMC, 2333 ZC Leiden, The Netherlands; Leiden Computational Biology Center, Biomedical Data Sciences, LUMC, 2333 ZC Leiden, The Netherlands; Leiden Computational Biology Center, Biomedical Data Sciences, LUMC, 2333 ZC Leiden, The Netherlands; Delft Bioinformatics Lab, Intelligent Systems, TU Delft, 2628 XE Delft, The Netherlands; Molecular Epidemiology, Biomedical Data Sciences, LUMC, 2333 ZC Leiden, The Netherlands; Molecular Epidemiology, Biomedical Data Sciences, LUMC, 2333 ZC Leiden, The Netherlands; Max Planck Institute for the Biology of Ageing, 50931 Cologne, Germany; Molecular Epidemiology, Biomedical Data Sciences, LUMC, 2333 ZC Leiden, The Netherlands; Leiden Computational Biology Center, Biomedical Data Sciences, LUMC, 2333 ZC Leiden, The Netherlands; Delft Bioinformatics Lab, Intelligent Systems, TU Delft, 2628 XE Delft, The Netherlands

## Abstract

**Motivation:**

^1^H-NMR metabolomics is rapidly becoming a standard resource in large epidemiological studies to acquire metabolic profiles in large numbers of samples in a relatively low-priced and standardized manner. Concomitantly, metabolomics-based models are increasingly developed that capture disease risk or clinical risk factors. These developments raise the need for user-friendly toolbox to inspect new ^1^H-NMR metabolomics data and project a wide array of previously established risk models.

**Results:**

We present MiMIR (Metabolomics-based Models for Imputing Risk), a graphical user interface that provides an intuitive framework for *ad hoc* statistical analysis of Nightingale Health’s ^1^H-NMR metabolomics data and allows for the projection and calibration of 24 pre-trained metabolomics-based models, without any pre-required programming knowledge.

**Availability and implementation:**

The R-shiny package is available in CRAN or downloadable at https://github.com/DanieleBizzarri/MiMIR, together with an extensive user manual (also available as Supplementary Documents to the article).

**Supplementary information:**

Supplementary data are available at *Bioinformatics* online.

## 1 Introduction

Quantitative high-throughput metabolomics data is steadily gaining popularity in epidemiological research, as exemplified by consortia like COMETS (Yu *et al.*, 2019), BBMRI-nl (van den Akker Erik *et al.*, 2020) and the UK-Biobanks (Nightingale Health UK Biobank Initiative *et al.*, 2021), accumulating ^1^H-NMR metabolomics data in ∼46 000, ∼26 000 and ∼120 000 individuals respectively. Combinations of metabolic features on measured by the Nightingale Health platform have previously been shown to be indicative of various disease risks: COVID severity (Nightingale Health UK Biobank Initiative *et al.*, 2021), Type2 Diabetes (Ahola-Olli *et al.*, 2019) and cardiovascular disease (Würtz *et al.*, 2015). Deelen *et al.* (2019) even derived a *mortality score* using this platform, which has a higher accuracy in predicting all-cause mortality, as compared to models of conventional risk factors. Moreover, we recently demonstrated that ^1^H-NMR metabolomics data can be used to impute ‘at risk status’ for 19 conventional clinical variables, and that these models can act as *metabolic surrogates* for their original values (Bizzarri *et al.*, 2022).

## 2 Materials and methods

As there is a growing interest in integrating metabolomics-based scores in epidemiological studies, we developed MiMIR (Metabolomics-based Models for Imputing Risk), a toolbox enabling visual inspection, rapid data exploration and easy projection of the pre-trained metabolic models on Nightingale Health ^1^H-NMR metabolomics data. We currently have included models trained to disease risks (e.g. COVID severity, mortality), and conventional risk factors (e.g. age, prevalent diabetes), with the intention to update the software with scores released in the future.

MiMIR is an interactive downloadable R-shiny web app that runs locally, and thus is compliant with current GDPR regulations. It guides users through simple steps to obtain 24 metabolomics-based scores suitable for downstream epidemiological analyses, using metabolite quantifications created by Nightingale Health. The analyses available in MiMIR are grouped in four main sections:

*Data exploration:* A user can upload Nightingale Health ^1^H-NMR metabolomics data as well as associated phenotypic variables (optionally uploaded). With MiMIR, distributions, completeness and correlations of all uploaded variables can be explored and compared to their distributions in ∼26 000 samples from BBMRI.nl.

*Pre-processing:* The pre-trained metabolic models in the app follow the pre-processing and transformation of the metabolomics data, as outlined in their respective publications. MiMIR runs these different procedures in parallel, allowing for a faithful derivation of each score. Generally, these steps consist of sample selection, normalization and imputation.

*Metabolic surrogates*: MiMIR calculates *metabolic surrogates* that represent the probability of a sample to be at risk for 19 variables clinically used in epidemiological studies (Bizzarri *et al.*, 2022). The surrogate scores can be calibrated using Platt Calibration (Platt, 1999), which can be evaluated visually (reliability diagrams) or statistically summarized (ECE, MCE and decrease log-loss) using a holdout set of 20% of the uploaded data (Supplementary Materials S5.1.4). Area Under the ROC Curves (AUCs) are calculated for the scores if their corresponding phenotypic variable is available and compared to a Leave One Biobank Out Validation (LOBOV) performed within BBMRI-nl (Bizzarri *et al.*, 2022).

*Analysis*: All 24 metabolomics-based scores, surrogates and risk models, can be subjected to exploratory association analyses. For instance, scores can be mutually correlated, associated with individual metabolites, or with phenotypic variables of interest, or used as potential confounders. Results can be comprehensively visualized, for instance, Kaplan Meier plots can be used to examine the ability of the metabolic scores to predict events over time.

## 3 Results

**Fig. 1.**
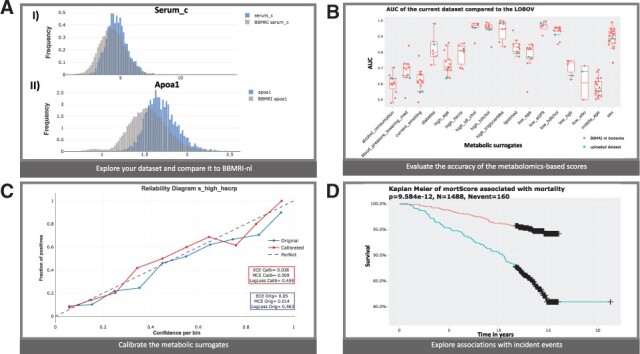
Application in the Leiden Longevity Study: (**A**) distributions of (I) serum_c and (II) apoa1 in LLS_PAROFFs (blue) and BBMRI-nl (grey); (**B**) AUC of the 19 surrogate clinical variables in the uploaded dataset (blue) compared with the results of the LOBOV (red); (**C**) Calibration of the surrogate for high hscrp (blue = uncalibrated, red = calibrated); (**D**) Kaplan Meier for mortality comparing people in the higher (blue) and lower (red) tertiles of the mortality score (A color version of this figure appears in the online version of this article.)

We illustrate the use of MiMIR with data assayed in a middle-aged subset of the Leiden Longevity Study (LLS_PAROFFs = 2307 individuals, median age = 59 y.o.) (Westendorp *et al.*, 2009). First, we evaluate the metabolomics features assayed by Nightingale Health through correlation plots and histograms of their distributions. When comparing these distributions with those from BBMRI-nl (Fig. 1A), a user can, for example, notice that serum total cholesterol (serum_c) and apoalipoprotein A1 (apoa1) show higher values in LLS_PAROFFs compared to BBMRI-nl (Fig. 1A).

A valuable feature of the scores obtained with MiMIR is that they can be used to impute unmeasured clinical variables. In case a clinical variable is at least partially measured, we can evaluate the accuracy of the imputations as generated by the surrogate models. As both, serum_c and apoa1, play a major role within the ‘lipid medication’ surrogate model, one can notice that the ‘lipid medication’ surrogate in LLS_PAROFF is less accurate, as compared to what is generally observed in BBMRI-nl (Bizzarri *et al.*, 2022) (Fig. 1B). In comparison, other *surrogates* perform better: ‘diabetes’ (AUC_LLS_PAROFF_=0.85, mean ${\mathrm{AUC}}_{\mathrm{LOBOV}}=0.8$), or similarly: ‘high hscrp’ (AUC_LLS_PAROFF_=0.811, mean ${\mathrm{AUC}}_{\mathrm{LOBOV}}=0.8$1), in LLS_PAROFFs than in other cohorts of BBMRI-nl (Fig. 1B).

Optionally, surrogate models can be calibrated in case some data on the respective clinical variables is at least partially available. A first glance to the reliability diagram of ‘high hscrp’ shows more appropriate probability distributions after applying Platt Calibration (Fig. 1C), with the calibrated values being closer to the diagonal in the reliability diagram and decreasing calibration errors (${\Delta \mathrm{ECE}}_{\left(\mathrm{calib}-\mathrm{orig}\right)}=\;-0.063$, ${\Delta \mathrm{MCE}}_{\left(\mathrm{calib}-\mathrm{orig}\right)}=\;-0.013$, ${\mathrm{LogLoss}}_{\left(\mathrm{calib}-\mathrm{orig}\right)}=\;-0.05$) in LLS_PAROFFs.

MiMIR allows to further explore the association of the metabolic features with the metabolomics-based scores or other phenotypic characteristics. For instance, 110 metabolic features are (individually) significantly associated with diabetes in LLS_PAROFFs, even after correcting for age, sex and the surrogate ‘obesity’, with glucose being the most significant (Supplementary Fig. S44). Longitudinal data can also be inspected by examining Kaplan–Meier curves. Although some metabolomics-based surrogates show association with mortality (e.g. ‘high hscrp’, Supplementary Fig. S42), one can appreciate that the mortality score is still the best score in predicting mortality (Fig. 1D).

## 4 Conclusion

MiMIR is a unique interactive R-shiny application that ties into the wealth of published ^1^H-NMR metabolomics-based models being released nowadays. Its potentiality comes from: (i) being able to inspect distributions of measurements in the context of other datasets, (ii) calculation and application of previously published metabolomics-based scores and (iii) imputation of clinically relevant variables in cohorts where this data is not readily present using metabolic surrogates. Due to its user-friendly interface, MiMIR empowers epidemiologists to inspect and analyze their own metabolic measurements and easily integrate these scores in their downstream analyses.

## Supplementary Material

btac388_Supplementary_Data
