# Supplementary material for: MiMIR: R-shiny application to infer risk factors and endpoints from Nightingale Health’s 1H-NMR metabolomics data
Source: Bioinformatics. 2022 Jun 13;38(15):3847–9. doi: 10.1093/bioinformatics/btac388 (PMC9344846; doi:10.1093/bioinformatics/btac388)
Supplement: btac388_Supplementary_Data [file btac388_supplementary_data.docx]

Application Note Supplementary Information

MiMIR: R-shiny application to infer risk factors and endpoints from ^1^H-NMR Metabolomics data

D. Bizzarri^1,2^, M.J.T. Reinders^2,3^, M. Beekman^1^, P.E. Slagboom^1,4^ and E.B. van den Akker^1,2,3, #^

^1^ Molecular Epidemiology, LUMC, Leiden, The Netherlands

^2^ Leiden Computational Biology Center, LUMC, Leiden, The Netherlands

^3^ Delft Bioinformatics Lab, TU Delft, Delft, The Netherlands

^4^ Max Planck Institute for the Biology of Ageing, Cologne, Germany

**Index**

[1. Description 2](#_Toc91064385)

[2. Supplementary document 1 - Manual 4](#_Toc91064386)

[2.1. Start the application: MiMIR 4](#_Toc91064387)

[2.2. Tab 1: Upload datasets 6](#_Toc91064388)

[2.2.1. Example dataset 6](#_Toc91064389)

[2.2.2. Upload your files 6](#_Toc91064390)

[2.3. Tab 2: “Your dataset” 13](#_Toc91064391)

[2.3.1. Metabolites 13](#_Toc91064392)

[2.3.2. Binary Phenotypes 15](#_Toc91064393)

[2.4. Tab 3: Settings QC (OPTIONAL) 18](#_Toc91064394)

[2.4.1. Pre-processing MetaboAge 18](#_Toc91064395)

[2.4.2. Preprocessing Metabolic surrogates 18](#_Toc91064396)

[2.4.3. Pre-processing other scores 18](#_Toc91064397)

[2.5. Predicted Scores 19](#_Toc91064398)

[2.5.1. Tab 4: Surrogates 19](#_Toc91064399)

[2.5.2. Tab 5: Metabolomics-scores 25](#_Toc91064400)

[2.6. Download Results 30](#_Toc91064401)

[3. Supplementary document 2 - Application to the Leiden Longevity study 31](#_Toc91064402)

[3.1. Study Description 31](#_Toc91064403)

[3.2. Acknowledgements 31](#_Toc91064404)

[3.3. Ethics statement 31](#_Toc91064405)

[3.4. Metabolic features 31](#_Toc91064406)

[3.5. Metabolic surrogates 33](#_Toc91064407)

[3.6. Metabolic scores 35](#_Toc91064408)

[4. Supplementary document 3 – The synthetic dataset 38](#_Toc91064409)

[5. References 43](#_Toc91064410)

# Description

This R-shiny webtool was built to facilitate user-friendly analyses of ^1^H-NMR metabolomics data assayed by Nightingale Health. It aims to support a quick and easy projection, evaluation and/or calibration of 24 pre-trained multivariate models in epidemiological data sets. Obtained scores can be used to enrich epidemiological studies with additional information that can be easily incorporated in downstream analyses. Of note, projected models were generally trained in large studies and are often reliant on scaling the metabolites. Consequentially, we strongly advice to derive scores with our app only in studies with a sufficient sample size (N>30), and not in small groups of samples. Projection of models should not be performed on a single sample. This app is designed to project all available models on a single dataset each time, after which they can be downloaded in a single data sheet. At the moment of writing the app allows for projection of the following metabolic models:

1. Mortality_score: a multivariate model predicting all-cause mortality [1]. It is based on 14 metabolic features and trained using a stepwise Cox regression analysis in a meta-analysis on 12 cohorts composed by 44,168 individuals.
2. MetaboAge: a multivariate model indicating the biological age of an individual, based on 56 metabolic features [2]. It was trained using a linear regression in BBMRI-nl, a Consortium of 28 cohorts comprising ~25,000 individuals. Another age-related variable is obtained from it:

- Delta MetaboAge: Difference between MetaboAge and chronological age.

1. Surrogate clinical variables: 19 multivariate scores indicating a range of binary clinical variables [3]. All the models were trained using logistic ElasticNET regressions in BBMRI-nl using 56 metabolic features. The surrogate metabolic markers are:

- s_sex (surrogate sex) towards 1 indicates a male (s_sex=1 if male).
- s_diabetes (surrogate diabetes) towards 1 indicates prevalent type 2 diabetes status.
- s_metabolic_syndrome (surrogate metabolic syndrome) towards 1 indicates prevalent metabolic syndrome status.
- s_lipidmed (surrogate lipid medication) towards 1 indicates the use of statins.
- s_blood_pressure_lowering_med (surrogate blood pressure lowering medication) towards 1 indicates the use of anti-hypertensive medication.
- s_current_smoking (surrogate current smoking) towards 1 indicates current smoking behavior.
- s_alcohol_consumption (surrogate alcohol consumption) towards 1 indicates regular alcohol usage (s_alcohol_consumption=1 if drinking alcohol).
- s_high_age (surrogate high age) towards 1 indicates a person older than 65 years.
- s_middle_age (surrogate middle age) towards 1indicates a person whose age is between 45 years old and 65 years.
- s_low_age (surrogate low age) towards 1 indicates a person younger than 45 years.
- s_obesity (surrogate obesity) towards 1 indicates obesity.
- s_high_hscrp (surrogate high hscrp) towards 1 if a person has hsCRP levels exceeding the clinical threshold > 3mg/L.
- s_high_triglycerides (surrogate high triglycerides) towards 1 if a person has triglycerides levels exceeding the clinical threshold $\geq$ 2.3 mmol/L.
- s_high_ldl_chol (surrogate high ldl cholesterol) towards 1 if a person has LDL cholesterol levels exceeding the clinical threshold $\geq$ 4.1 mmol/L.
- s_high_totchol (surrogate high total cholesterol) towards 1 if a person has total cholesterol levels exceeding the clinical threshold $\geq$ 6.2 mmol/L.
- s_low_hdlchol (surrogate low HDL cholesterol) towards 1 if a person has HDL levels exceeding the clinical threshold $\leq$ 1.3 mmol/L.
- s_low_eGFR (surrogate low eGFR) toward 1 if a person has levels of estimated Glomerular Filtration Rate exceeding the clinical threshold $\leq$ 60 ml/min.
- s_low_wbc (surrogate low white blood cell counts) indicating if a person has levels of white blood cell counts exceeding the clinical threshold $\leq$ 4.5x${10}^{9}$ L.
- s_low_hgb (surrogate low hemoglobin) indicating if a person has low levels of hemoglobin exceeding the clinical threshold $\leq$ 6.67 mmol/L in men and 7.62 mmol/L in women.

1. COVID score: a multivariate model predicting the risk of severe COVID-19 infection [4]. It is based on 37 metabolic features and trained using LASSO regression on 52,573 samples from the UK-biobanks.
2. T2D score: A score associated with incident Type 2 Diabetes, made by Ahola-Olli et al [5]. It is constructed using sex, age, BMI, fasting glucose and 3 metabolic features. It was trained using a stepwise logistic regression on 3 cohorts.
3. CVD score: A multi biomarker score indicating cardiovascular risk, made by Würtz et al. [6]. It is based on sex, systolic blood pressure, current smoking, prevalent diabetes, antihypertensive medication, lipid lowering medication, total cholesterol, hdl cholesterol and 5 metabolic features. It was trained using a Cox proportional hazard regression model.

For a detailed description of the metabolic scores, please refer to the original manuscripts [1]–[6]. We will refer to each metabolic score with the nomenclature used in the list above.

MiMIR is created on a R-shiny framework, on a machine with R version 4.1.2. Most of the front-end images are created using the package plotly [7] to generate interactive plots, which can be easily downloaded. Using a R-markdown document that contains all the inputs and steps performed during the analysis we ensure reproducibility of the results.

The remainder of this document is divided in three different sections: [1] An extensively illustrated User Manual, containing all the information about how to use MiMIR. A synthetic dataset allows users to play with the functionality of the MiMIR package. [2] Application on the Leiden Longevity Study Partner Offspring [8]. [3] A description of the synthetic dataset.

# Supplementary document 1 - Manual

## Start the application: MiMIR

#### Pre-requisites

This package has a series of pre-requisites to be able to run:

- Install R ≥v4.1
- R-studio preferable
- A web browser Google Chrome preferably

This application is not granted to work with earlier versions of R.

#### Installation

Once these pre-requisites are satisfied the package can be installed. Open R-studio and type the following steps (also available at <https://github.com/DanieleBizzarri/MiMIR>):

1. Install the “devtools” package (if not already done).

| install.packages("devtools") |
| --- |

1. Install the “MiMIR” package from Github/DanieleBizzarri/MiMIR

| library("devtools")  devtools::install_github("DanieleBizzarri/MiMIR") |
| --- |

1. Launch the application

| library("MiMIR")  MiMIR::startApp() |
| --- |

Running the command MiMIR::startApp() will first initialize and then open the application on your default web browser. If you prefer you can use also MiMIR::startApp(launch.browser = FALSE) to launch the App in an Rstudio window.

If this was successful, you should see a graphical interface menu that looks like:


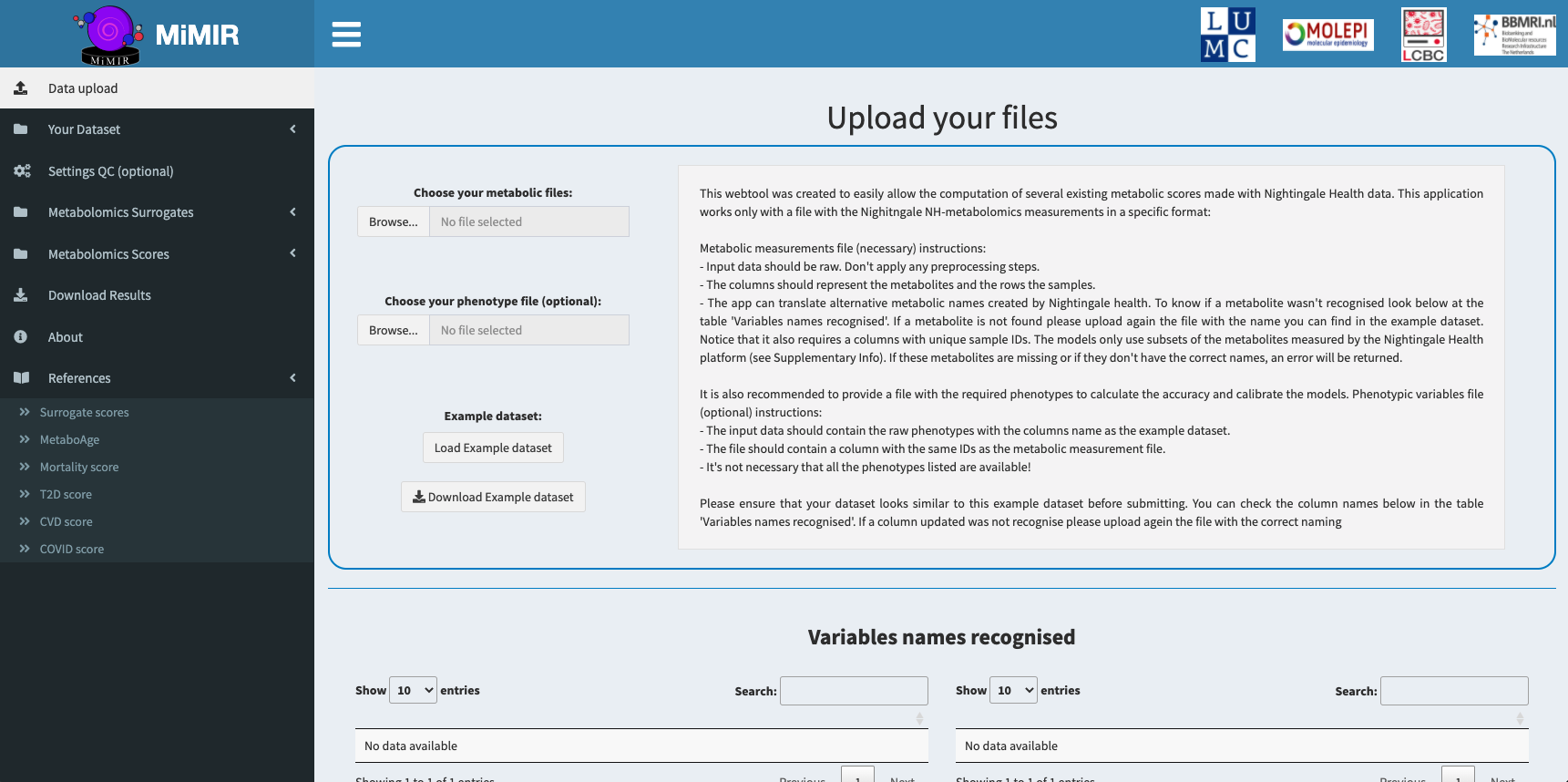


**Figure S1:** Application home page.

The Application is now ready to be used!

#### Installing packages manually

If there are some problems installing some packages, copy the line of a specific package into R/Rstudio console to install it manually”

| ## Shiny environment  if (!require("shiny")) install.packages("shiny")  if (!require("shinydashboard")) install.packages("shinydashboard")  if (!require("shinyWidgets")) install.packages("shinyWidgets")  if (!require("shinycssloaders")) install.packages("shinycssloaders")  if (!require("shinyjs")) install.packages("shinyjs")  #Statistics libraries  if (!require("DT")) install.packages("DT")  if (!require("foreach")) install.packages("foreach")  if (!require("glmnet")) install.packages("glmnet")  if (!require("matrixStats")) install.packages("matrixStats")  if (!require("plyr")) install.packages("plyr")  if (!require("stats")) install.packages("stats")  if (!require("reshape2")) install.packages("reshape2")  if (!require("caret")) install.packages("caret")  if (!require("BiocManager", quietly = TRUE)) install.packages("BiocManager")  if (!require("limma")) BiocManager::install("limma", force=TRUE)  if (!require("purrr")) install.packages("purrr")  if (!require("dplyr")) install.packages("dplyr")  if (!require("rmarkdown")) install.packages("rmarkdown")  #Imaging libraries  if (!require("pheatmap")) install.packages("pheatmap")  if (!require("RColorBrewer")) install.packages("RColorBrewer")  if (!require("pROC")) install.packages("pROC")  if (!require("plotly")) install.packages("plotly")  if (!require("heatmaply")) install.packages("heatmaply")  if (!require("ggplot2")) install.packages("ggplot2")  if (!require("ggfortify")) install.packages("ggfortify")  if (!require("survival")) install.packages("survival")  if (!require("survminer")) install.packages("survminer") |
| --- |

## Tab 1: Upload datasets

### Example dataset

We provide a synthetic dataset that can be used to explore MiMIR. This dataset can be directly loaded in the app by clicking the button “Load Example dataset” or it can be downloaded using the button “Download Example dataset”. In the latter case, MiMIR will generate a .zip file containing two files: one called “synthetic_metabolic_dataset” and the other “synthetic_phenotypic_dataset”, respectively containing a matrix of ${}^{1}{H-NMR}$metabolomics concentrations and a matrix with phenotypic information for a synthetic file obtained from the cohort LLS_PARTOFF [8].


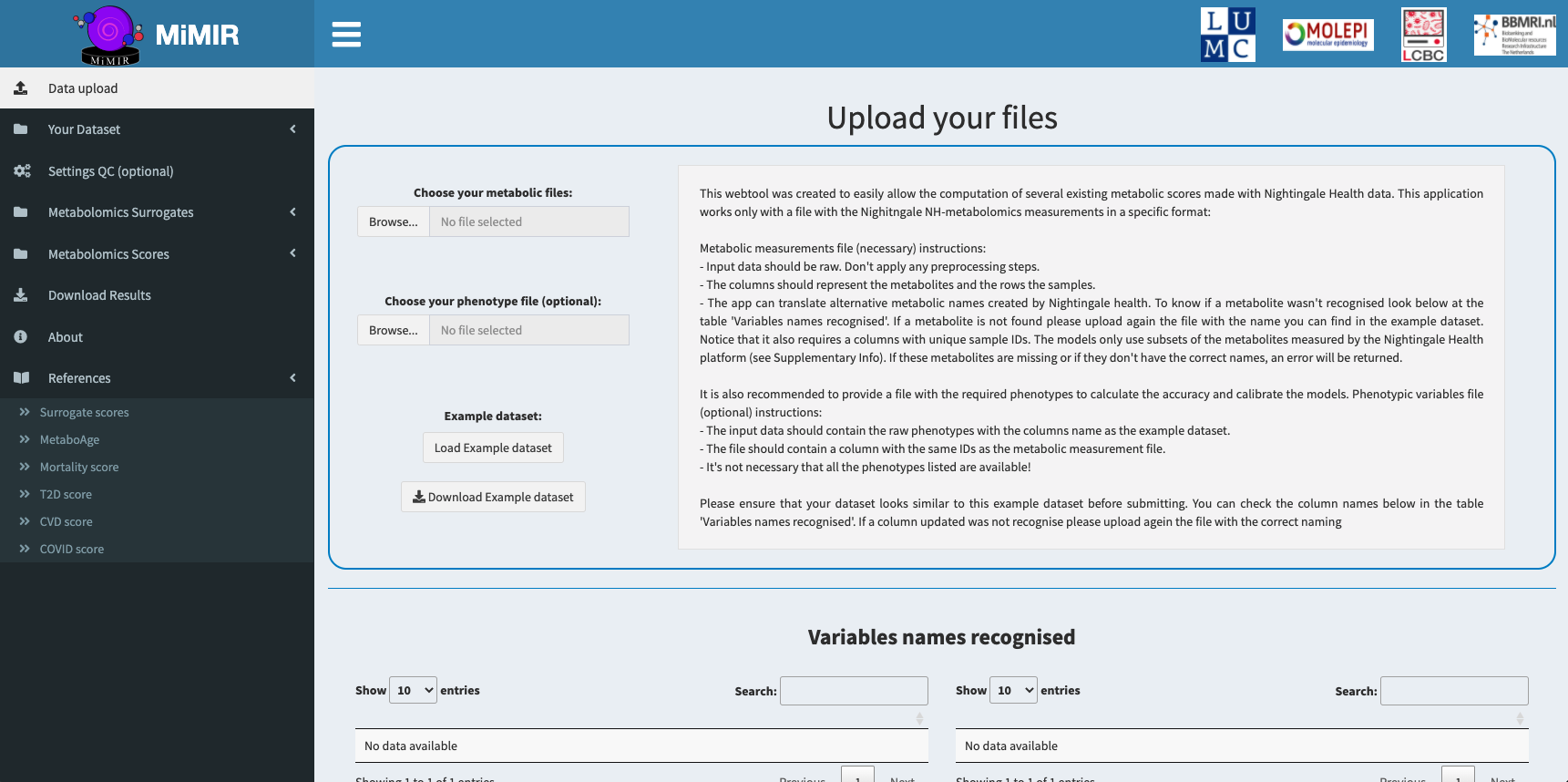


z

**Figure S2:** Button to Download the example dataset in the home page.

The synthetic dataset contains 500 samples whose metabolite levels and phenotype data were modeled after the Partner-Offspring generation (LLS-PO) of the Leiden Longevity Study (median age = 59 years) [8] and was created using the package “synthpop” [9]. For a detailed explanation of how the synthpop package was used to create the synthetic data we refer the reader to **Supplementary document 3** and to the paper by Nowok *et al.* [9].

Upload your files

Before uploading your own dataset in the application, please make sure:

1. You have local files in format .csv or .tsv.
2. the metabolic features are named like the features in the example dataset or with the names given by Nightingale Health.
3. the clinical variables (if available) are named like the features in the example dataset.
4. the two files have the same number of samples.
5. the first column of both files contains unique IDs for each sample.
6. the sample IDs are the same in the metabolomics file and the phenotypic file.
7. you didn’t apply preprocessing (i.e. normalize, etc.) to the data.
8. The missing values are encoded as NA.


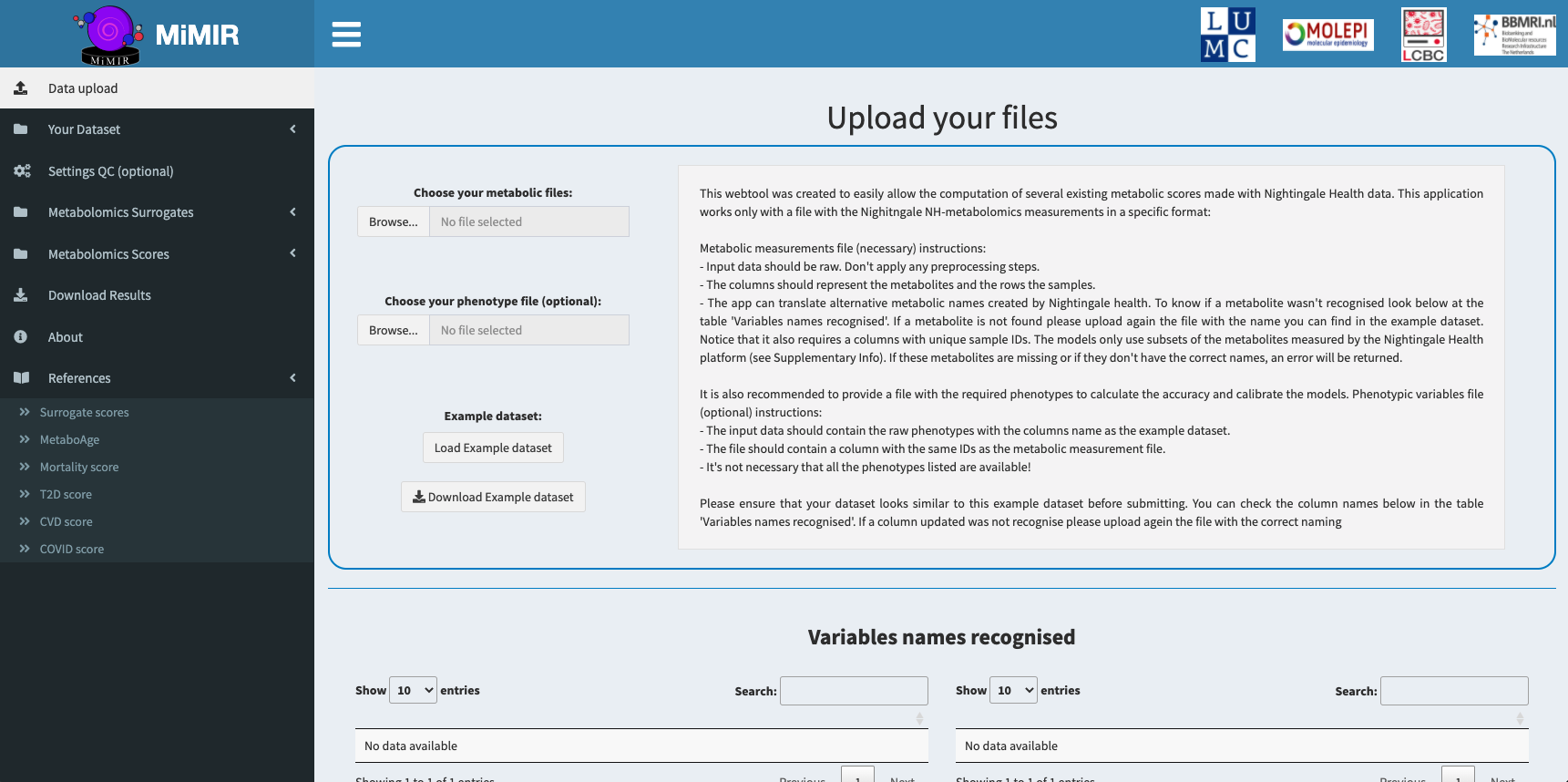


Metabolites concentration file

Phenotypic variables file

**Figure S3:** Button to Browse the files with metabolic concentration and phenotypic variables

Both the ${}^{1}{H-NMR}$ metabolomics and phenotypic files can be visualized in the tables at the bottom of the “Data upload” tab.

#### Metabolites concentration file

When the metabolomics concentration are uploaded, MiMIR will try to extract the metabolic features needed to calculate the scores, as named in the column names. MiMIR can recognize several alternative names of the features that are used by Nightingale Health, using a translation file similar to the one created for the R package “ggforestplot” [10]. MiMIR will provide an overview of all required metabolic features, and displays metabolic features that were not recognized in red at the top of the list (***Figure S4***). In this case you will need to check the names of the metabolic features in red and substitute them with a nomenclature that can be recognized by MiMIR (look at the example file, and Table 1). If all metabolic features are in green the dataset was correctly uploaded, and the user can continue with the analyses.


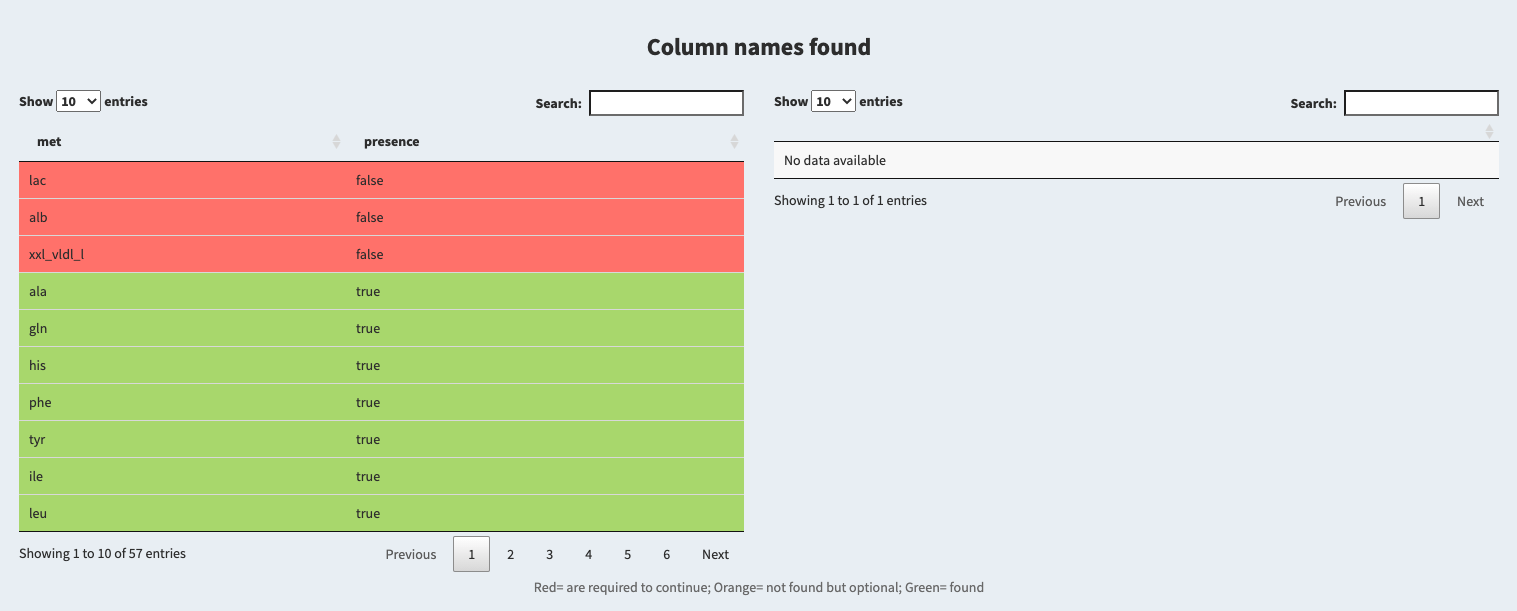


**Figure S4:** List with the metabolites needed to use the pre-trained models. MiMIR didn’t find the variables “lac”, “alb” and “xxl_vldl_l” in the current dataset, therefore are in red. If MiMIR can’t find all the necessary variables it will not be able to continue the rest of the analyses.

Table 1: Table describing the metabolic features collected by Nightingale necessary to run MiMIR. Under “Metabolic feature names” you can find the names of the features that are used by MiMIR, in “Description” you can see a short description of what metabolites the features are representing, in unit you can see the unit of measure and lastly in which pre-trained models they are included.

| Metabolic feature names | Description | Unit | Metabolic score contribution |
| --- | --- | --- | --- |
| m_vlvl_l | Total lipids in medium VLDL | mmol/l | MetaboAge and surrogates |
| s_vldl_l | Total lipids in small VLDL | mmol/l | MetaboAge and surrogates |
| xs_vldl_l | Total lipids in very small VLDL | mmol/l | MetaboAge and surrogates |
| idl_l | Total lipids in IDL | mmol/l | MetaboAge and surrogates |
| idl_c | Total cholesterol in IDL | mmol/l | MetaboAge and surrogates |
| l_ldl_l | Total lipids in large LDL | mmol/l | MetaboAge and surrogates |
| m_ldl_l | Total lipids in medium LDL | mmol/l | MetaboAge and surrogates |
| m_hdl_l | Total lipids in medium HDL | mmol/l | MetaboAge and surrogates |
| s_hdl_l | Total lipids in small HDL | mmol/l | Mortality score, MetaboAge, surrogates |
| s_ldl_l | Total lipids in small LDL | mmol/l | MetaboAge and surrogates |
| vldl_d | Mean diameter for VLDL particles | nm | Mortality score, MetaboAge, surrogates |
| ldl_d | Mean diameter for LDL particles | nm | MetaboAge and surrogates |
| hdl_d | Mean diameter for HDL particles | nm | MetaboAge and surrogates |
| serum_c | Serum total cholesterol | mmol/l | MetaboAge and surrogates |
| vldl_c | Total cholesterol in VLDL | mmol/l | MetaboAge and surrogates |
| ldl_c | Total cholesterol in LDL | mmol/l | MetaboAge and surrogates |
| hdl_c | Total cholesterol in HDL | mmol/l | MetaboAge and surrogates |
| hdl2_c | Total cholesterol in HDL2 | mmol/l | MetaboAge and surrogates |
| hdl3_c | Total cholesterol in HDL3 | mmol/l | MetaboAge and surrogates |
| serum_tg | Serum total triglycerides | mmol/l | MetaboAge and surrogates |
| totpg | Total phosphoglycerides | mmol/l | MetaboAge and surrogates |
| pc | Phosphatidylcholine and other cholines | mmol/l | MetaboAge and surrogates |
| sm | Sphingomyelins | mmol/l | MetaboAge and surrogates |
| totcho | Total cholines | mmol/l | MetaboAge and surrogates |
| apoa1 | Apolipoprotein A-I | g/l | MetaboAge and surrogates |
| apob | Apolipoprotein B | g/l | MetaboAge and surrogates |
| totfa | Total fatty acids | mmol/l | MetaboAge and surrogates |
| unsatdeg | Estimated degree of unsaturation |  | MetaboAge and surrogates |
| dha | 22:6, docosahexaenoic acid | mmol/l | MetaboAge, surrogates, CVD score, COVID score |
| la | 18:2, linoleic acid | mmol/l | MetaboAge and surrogates |
| faw3 | Omega-3 fatty acids | mmol/l | MetaboAge and surrogates |
| faw6 | Omega-6 fatty acids | mmol/l | MetaboAge and surrogates |
| pufa | Polyunsaturated fatty acids | mmol/l | MetaboAge, surrogates, CVD score |
| mufa | Monounsaturated fatty acids; 16:1, 18:1 | mmol/l | MetaboAge, surrogates, COVID score |
| sfa | Saturated fatty acids | mmol/l | MetaboAge and surrogates |
| faw3fa | Ratio of omega-3 fatty acids to total fatty acids | % | MetaboAge and surrogates |
| faw6_fa | Ratio of omega-6 fatty acids to total fatty acids | % | MetaboAge and surrogates |
| pufa_fa | Ratio of polyunsaturated fatty acids to total fatty acids | % | Mortality score, MetaboAge, surrogates |
| mufa_fa | Ratio of monounsaturated fatty acids to total fatty acids | % | MetaboAge, surrogates |
| sfa_fa | Ratio of saturated fatty acids to total fatty acids | % | MetaboAge, surrogates, CVD score |
| glc | Glucose | mmol/l | Mortality score, MetaboAge, surrogates |
| lac | Lactate | mmol/l | Mortality score, MetaboAge, surrogates |
| cit | Citrate | mmol/l | MetaboAge and surrogates |
| ala | Alanine | mmol/l | MetaboAge and surrogates |
| gln | Glutamine | mmol/l | MetaboAge and surrogates |
| his | Histidine | mmol/l | Mortality score, MetaboAge, surrogates |
| ile | Isoleucine | mmol/l | Mortality score, MetaboAge, surrogates, COVID score |
| leu | Leucine | mmol/l | Mortality score, MetaboAge, surrogates |
| val | Valine | mmol/l | Mortality score, MetaboAge, surrogates |
| phe | Phenylalanine | mmol/l | Mortality score, MetaboAge, surrogates, T2D score by Ahola-Olli, CVD score |
| tyr | Tyrosine | mmol/l | MetaboAge, surrogates, COVID score |
| ace | Acetate | mmol/l | MetaboAge and surrogates |
| acace | Acetoacetate | mmol/l | Mortality score, MetaboAge, surrogates |
| crea | Creatinine | mmol/l | MetaboAge, surrogates, COVID score |
| alb | Albumin | signal area | Mortality score, MetaboAge, surrogates |
| gp | Glycoprotein acetyls, mainly a1-acid glycoprotein | mmol/l | Mortality score, MetaboAge, surrogates, COVID score |
| xxl_vldl_l | Total lipids in extremely large VLDL | mmol/l | Mortality score |
| l_vldl_ce_percentage | Cholesteryl esters in chylomicrons and extremely large VLDL | mmol/l | T2D score by Ahola-Olli |
| l_hdl_fc | Free cholesterol in large LDL | mmol/l | T2D score by Ahola-Olli |
| apob_apoa1 | Ratio of apolipoprotein B to apolipoprotein A1 | ratio | COVID score |
| faw6_faw3 | Ratio of omega-6 fatty acids to omega-3 fatty acids | ratio | COVID score |
| glycine | Glycine | mmol/l | COVID score |

#### Phenotypic variable file

The phenotypic variable file includes the phenotypic information that the user finds useful for the analyses. It is used to get estimations of the accuracy of the pre-trained models, to calibrate the surrogate models, but also partially to calculate the T2D score [5] and the CVD score, which are not exclusively based on metabolic features. Therefore, this is an optional file, but uploading this data will allow the user to use MiMIR to its full potential. The variables that should be included in this file are listed in Table 2, with the appropriate nomenclature. MiMIR will also report a list with the variables found in the uploaded dataset like the metabolic features. In this case the variables names which aren’t recognized are indicated with orange (Figure S5), because they are not essential for the rest of the analyses.

The missing values in this file, like in the metabolomics file should be encoded as NA.


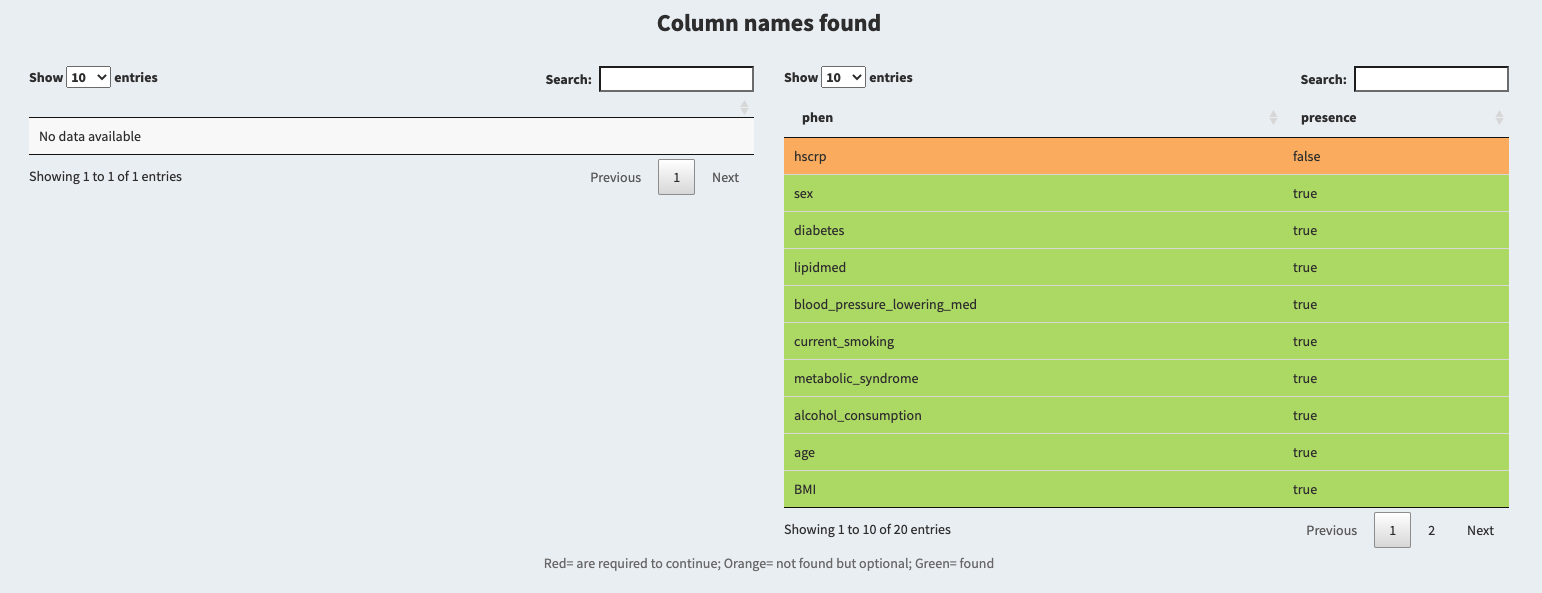


**Figure S5:** List with the phenotypes that were found in the uploaded dataset. MiMIR didn’t find the variables “hscrp” in the uploaded dataset. That means that it won’t be able to produce accuracy and calibration analyses, but it will be able to continue running.

The phenotypic variables and the values accepted by MiMIR are described in Table 2. Some variables are obtained as composite variables: 1) LDL cholesterol, is calculated using hdl cholesterol, triglycerides levels and total cholesterol using the Friedewald equation [11]; 2) eGFR (estimated Glomerular Filtration Rate), is calculated using age, sex and creatinine levels (included in the Nightingale Health platform), using the creatinine-based CKD-EPI equation [12]; 3) obesity, which is a binary variable describing if a person is clinically obese or not variable that uses BMI, waist circumference and sex [13]; 4) low_hgb (low hemoglobin), which is a binary variables describing ‘at risk’ levels of hemoglobin by using hemoglobin and sex [14]. In case the phenotypic file already includes BMI, LDL cholesterol or eGFR MiMIR won’t calculate them, but use uploaded ones.

**Table 2: Phenotypic variables:** Table describing the phenotypic variables that will be accepted by MiMIR. The column variable accepted represents the nomenclature for the column names of the phenotypic table. The column Description indicates what the columns represent. The column Values what values are accepted per each variable.

| Variables accepted | Description | Values |
| --- | --- | --- |
| sex | Sex | men: 1 or male,  women: 0 or female |
| diabetes | Prevalent diabetes status | TRUE: 1 or TRUE  FALSE: 0 or FALSE |
| lipidmed | Prevalent statins usage | TRUE: 1 or TRUE  FALSE: 0 or FALSE |
| blood_pressure_lowering_med | Prevalent antihypertensives usage | TRUE: 1 or TRUE  FALSE: 0 or FALSE |
| current_smoking | Current smoking | TRUE: 1 or TRUE  FALSE: 0 or FALSE |
| metabolic_syndrome | Prevalent metabolic syndrome | TRUE: 1 or TRUE  FALSE: 0 or FALSE |
| alcohol_consumption | Current alcohol consumption | TRUE: 1 or TRUE  FALSE: 0 or FALSE |
| age | Chronological age | Continuous (years) |
| BMI | Body Mass Index | Continuous (kg/cm^2^) |
| weight | weight | Continuous (kg) |
| height | height | Continuous (cm) |
| waist_circumference | Waist circumference | Continuous (cm) |
| hscrp | hsCRP | Continuous (mg/L) |
| triglycerides | triglycerides | Continuous (mmol/L) |
| ldl_chol | LDL cholesterol | Continuous (mmol/L) |
| hdlchol | HDL cholesterol | Continuous (mmol/L) |
| totchol | Total cholesterol | Continuous (mmol/L) |
| eGFR | Estimated glomerular filtration rate | Continuous (ml/min) |
| wbc | White blood cells | Continuous (L) |
| hgb | hemoglobin | Continuous (mmol/L) |
| glucose | Glucose | Continuous |
| systolic_blood_pressure | Systolic blood pressure | Continuous (mmHg) |
| Event | State of an event until censoring | 1 or TRUE if event observed  0 or FALSE if censored |
| EventAge | The age of the subject at the censoring | Continuous (years) |

Most of these phenotypic variables will also be used to evaluate and calibrate the metabolic surrogates [15]. The surrogates were trained to predict binary variables that were obtained using thresholds for “*at risk*” levels of generally used clinical variables. MiMIR is going to calculate the binary variables from the file that the user uploads. It is possible to inspect the binarized clinical variables at the bottom of the upload tab. Table 3 is a list with the variables and the thresholds used to obtain them.

**Table 3: Binary clinical variables:** Table describing the phenotypic variables that will be used by MiMIR to evaluate the accuracy or to calibrate the surrogates. Original variables represent the nomenclature for the clinical variables and should be present in the phenotypic file, “thresholds” are the thresholds to binarize following the work of Bizzarri et al. and the last columns represent the names for the final binarized variables result.

| Original variables | thresholds | Binarized variable names |
| --- | --- | --- |
| sex |  | sex |
| diabetes |  | diabetes |
| lipidmed (lipid medication) |  | lipidmed |
| blood_pressure_lowering_med  (blood pressure lowering medication) |  | blood_pressure_lowering_med |
| current_smoking |  | current_smoking |
| metabolic_syndrome |  | metabolic_syndrome |
| alcohol_consumption |  | alcohol_consumption |
| age | age$\geq$65 y.o. | high_age |
| age | 45 y.o. $\geq$ age < 65 y.o. | middle_age |
| age | age < 45 y.o. | low_age |
| BMI (or $\frac{\boldsymbol{wieght}}{\boldsymbol{height}^{\boldsymbol{2}}}$) | BMI$\geq$30 kg/$m^{2}$ and w.c. $\geq$ 102 cm [Male]  BMI$\geq$30 kg/$m^{2}$ and w.c. $\geq$ 93 cm [Female] [13] | obesity |
| waist_circumference |  |  |
| sex |  |  |
| hscrp | hscrp > 3mg/L | high_hscrp |
| triglycerides | trig$\geq$ 2.3 mmol/L [16] | high_triglycerides |
| ldl_chol  (ldl cholesterol) | ldl_chol $\geq$ 4.1 mmol/L [16] | high_ldl_chol |
| hdlchol  (hdl cholesterol) | hdlchol $\leq$ 1.3 mmol/L [16] | high_hdl_chol |
| totchol  (total cholesterol) | totchol $\geq$ 6.2 mmol/L [16] | high_totchol |
| eGFR (or using age, sex and Nightingale creatinine in the CKD EPI formula) | eGFR$\leq$ 60 ml/min [17] | low_eGFR |
| wbc  (white blood cells) | wbc$\leq$ 4.5x${10}^{9}$ L [14] | low_wbc |
| hgb  (hemoglogin levels) | hgb $\leq$ 6.67 mmol/L [Male]  hgb $\leq$ 7.62 mmol/L [Female] [14] | low_hgb |

## Tab 2: “Your dataset”

This tab was implemented to be able to have a quick and easy-to-use analysis of the uploaded dataset. All the Figures shown from this point on were realized using the synthetic dataset that is shipped with MiMIR, therefore any user should be able to reproduce them. Although this is fine for illustrative purposes, we do note that in general the performances on real data outperform those on synthetic data.

### Metabolites

In this section it is possible to perform some standard analyzes on the Nightingale Health metabolomics panel. The total amount of metabolic variables collected by Nightingale Health is 226 for EDTA plasma samples. This includes ratios and derived measurements, but all the metabolic scores uses a total of 65 features, which were selected to be the most uncorrelated and available during in the training datasets [6], [18]. [Table 1](#Table1) includes all the metabolites included in the scores. MiMIR allows the user to analyze this set of features in several ways:

1. Correlation heatmaps: it visualizes the Pearson correlations of the metabolites needed to calculate each metabolomics-based score. High correlations are indicated in red and low correlations in blue. This plot can help the user to understand the relations between the metabolic features and divide the features in highly correlated groups. Using the Radio Button on the side it’s possible to view the subsets of metabolites used for each of the scores.


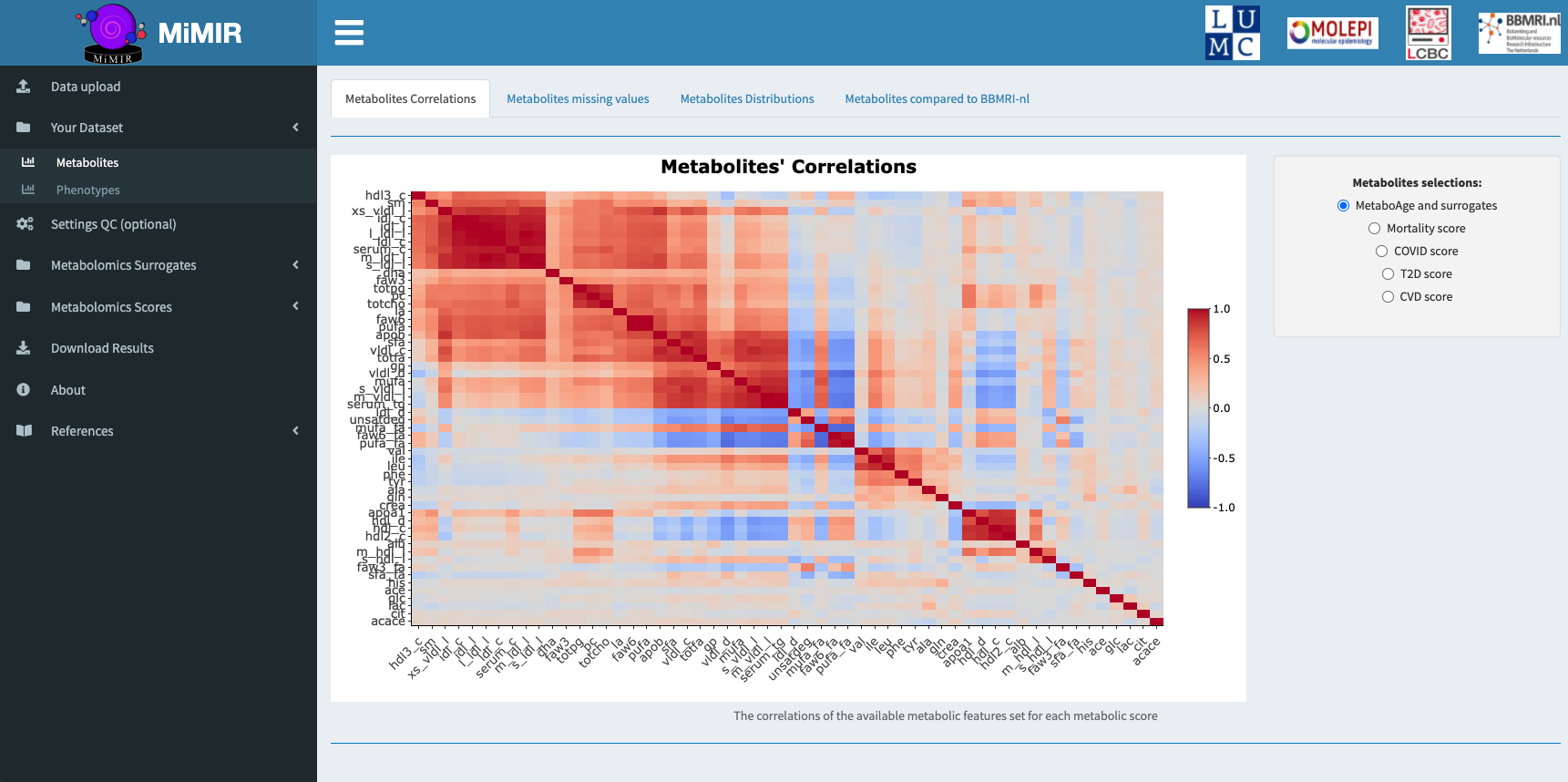


**Figure S6:** Correlation of the metabolites of the set of metabolites used for the metabolic surrogates and MetaboAge. The plot suggests that there are several groups of highly correlated features within the selection.

1. Metabolites missing values: This tab contains a heatmap indicating the available values in grey and missing in white. It also includes two bar plots on the sides: one to show the missingness per sample and another to show the missingness per metabolite. Also in this case it’s possible to limit the metabolites used for each of the scores .


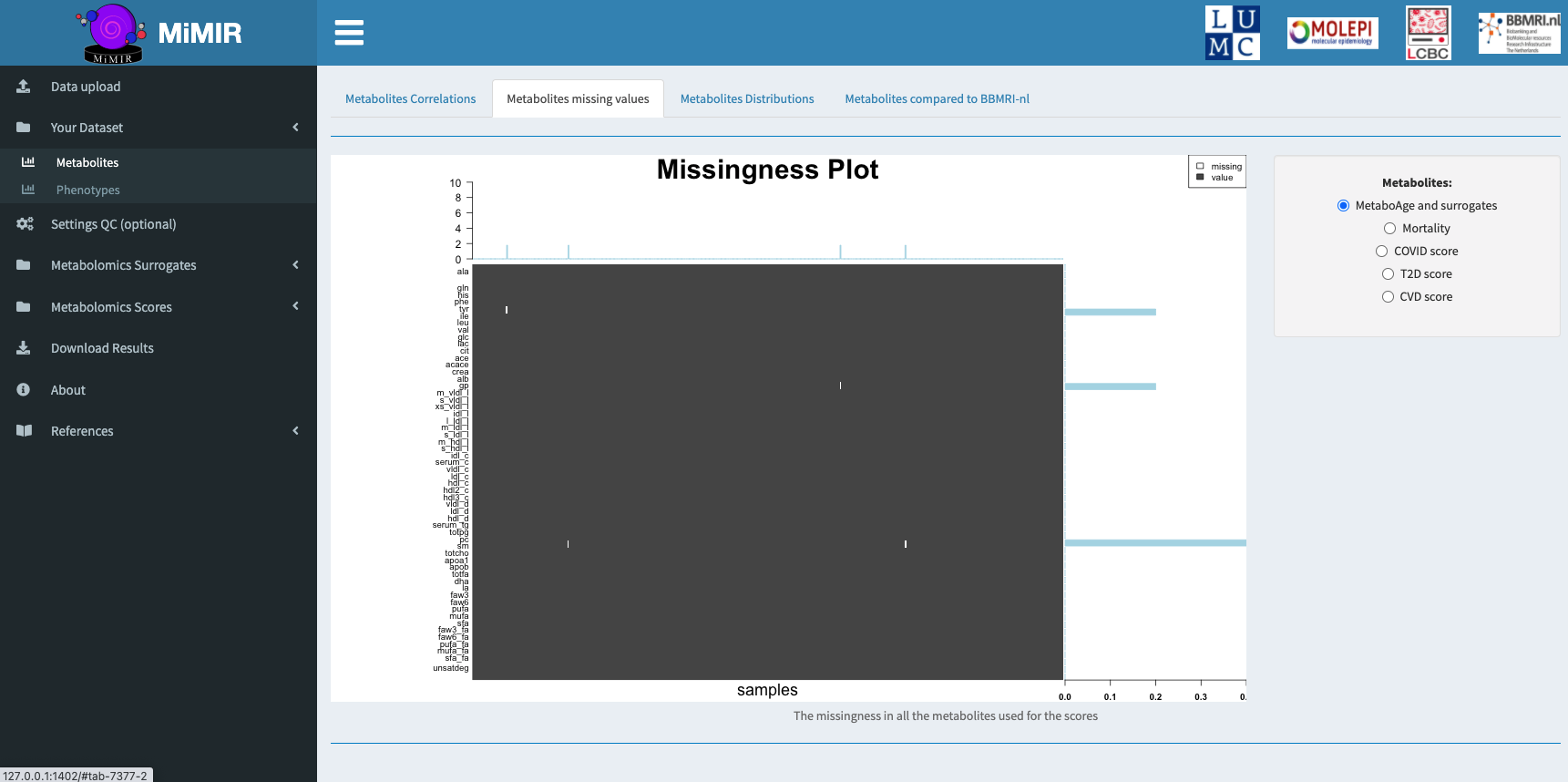


**Figure S7:** Missingness in the metabolic features used to calculate the COVID score. Glycine, one of the metabolic features necessary to calculate this score is completely missing in the dataset. Therefore, MiMIR won’t be able to calculate the COVID score. Using the radio buttons on the right it is possible to visualize the set of metabolites used in each score.

1. Metabolites Distributions: this interactive bar plot shows the distributions of one or more metabolic feature within the uploaded dataset. Using the Radio Button on the right, the user can visualize their z-scaled version, to allow for a better comparison.

| **[A]**  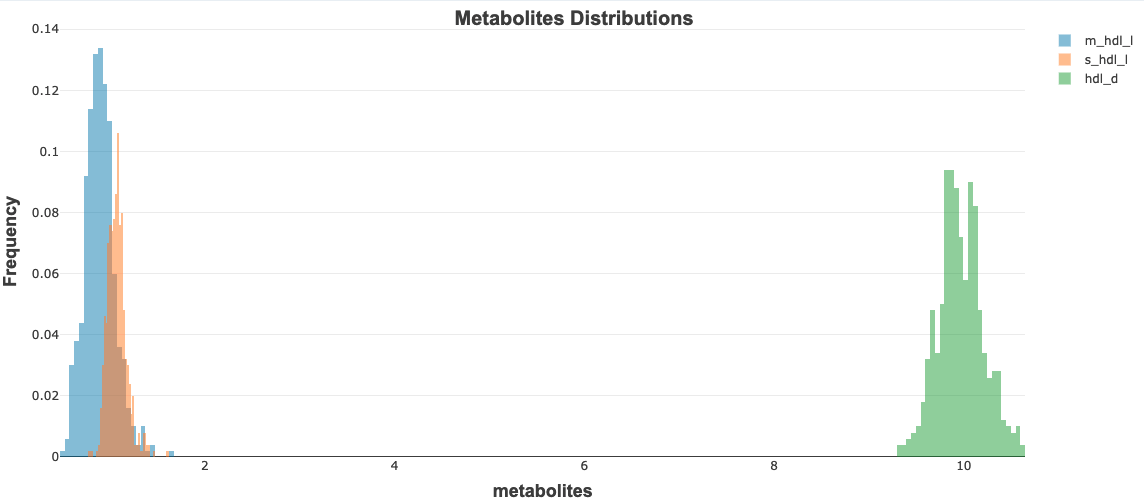 | **[B]**  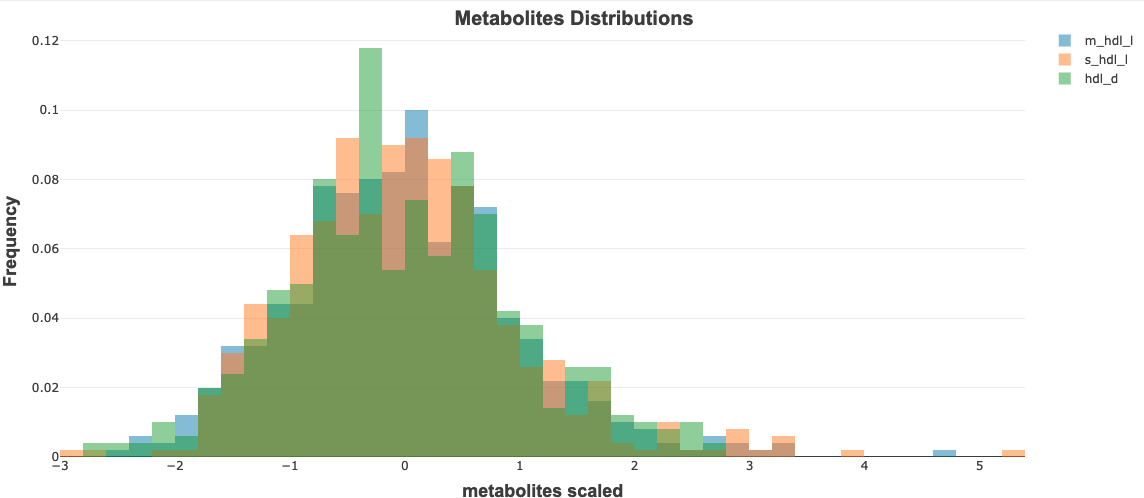 |
| --- | --- |

**Figure S8:** [A] Metabolites distributions of m_hdl_l, s_ldl_l and hdl_d and [B] their scaled version. In this picture we can notice the importance of the scaling to compare the features in the Nightingale platform. It is possible to select the metabolites to visualize with the check box and to scale with the radio button on the right side.

1. Metabolic distributions compared to BBMRI-nl: The distributions of metabolic feature in the uploaded dataset can be compared to the respective distributions observed in BBMRI-nl. On the bottom you can see also the relative importance of the metabolic features to each score. The relative importance of each of the metabolites for a particular model is calculated as its model coefficient divided by the total sum over all model coefficients in that model. In this way it is possible to verify whether the metabolites that contribute most to the models have a distribution that is similar to those observed in to BBMRI-nl.


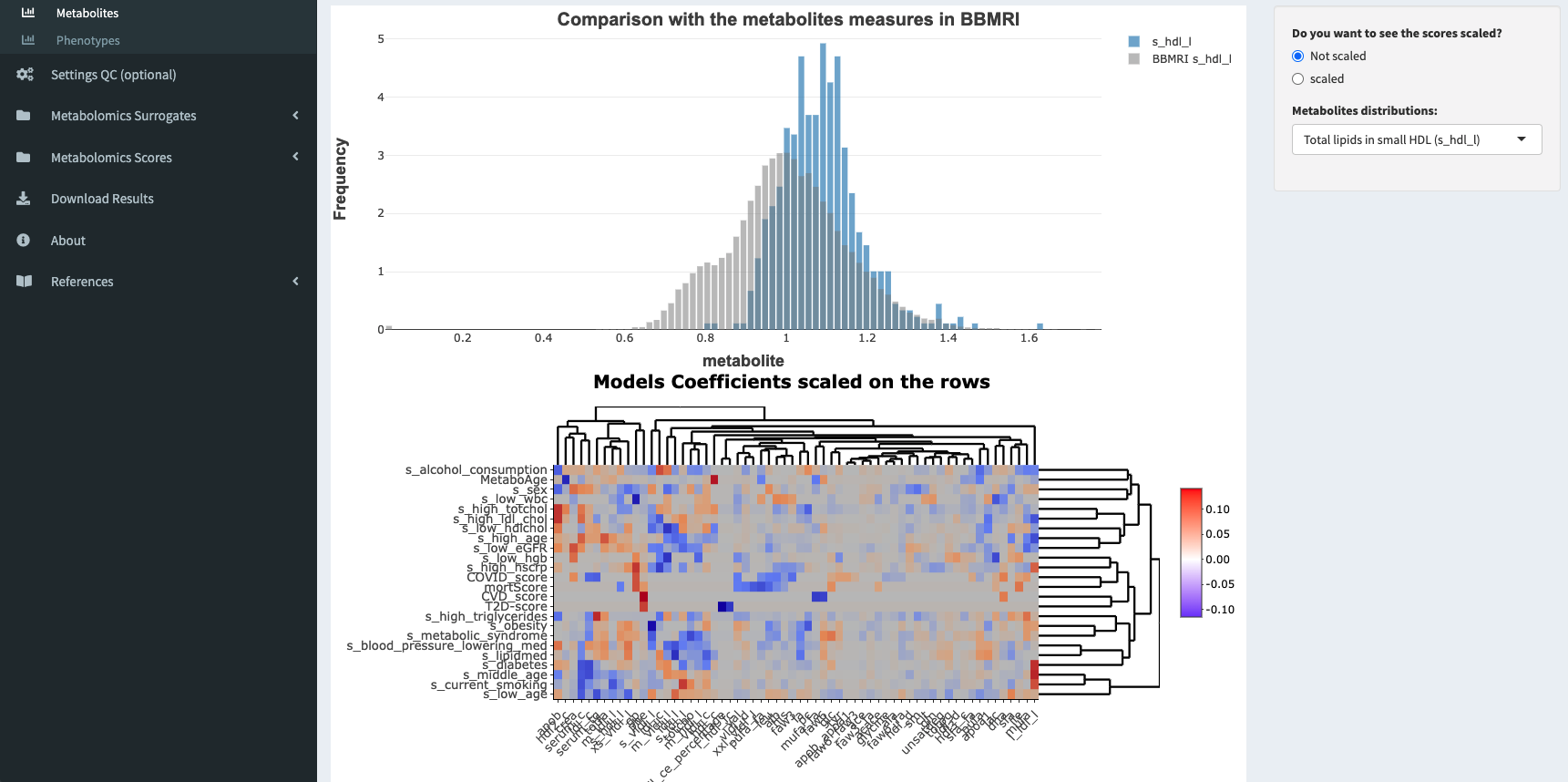


**Figure S9:** On the top, the distribution of s_hdl_l in LLS-PO compared to the distribution in BBMRI-nl, on the bottom the relative importance of the metabolic features in each model. We can see that s_hdl_l has a big impact on several metabolomics-based scores (e.g. ‘metabolic syndrome’).

### Binary Phenotypes

This section was created to analyze the phenotypic information. MiMIR allows one to calculate and analyse the binarized clinical variables described in section [2.2.2](#_Phenotypic_variable_file) and Table 3. The figures produced are:

1. Phenotypes summary: a table which collects all the summary statistics for the variables uploaded in the phenotypes file


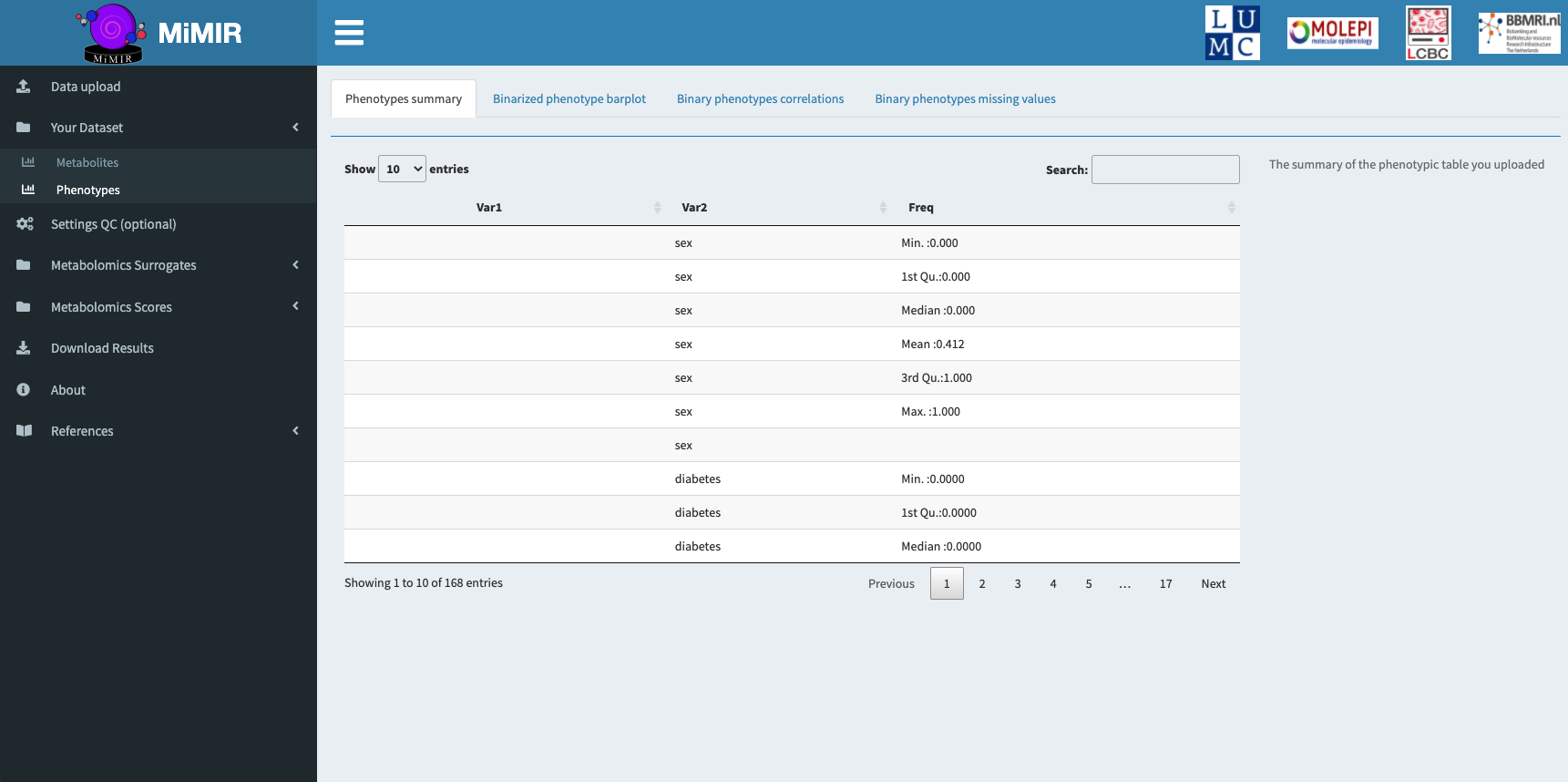


***Figure S10:*** The summary statistics of age and BMI in the synthetic dataset.

1. Binarized phenotypes bar plot: An interactive bar plot indicating the amount of 0/FALSE (in grey) and 1/TRUE (in blue) are present in the binary phenotypes obtained


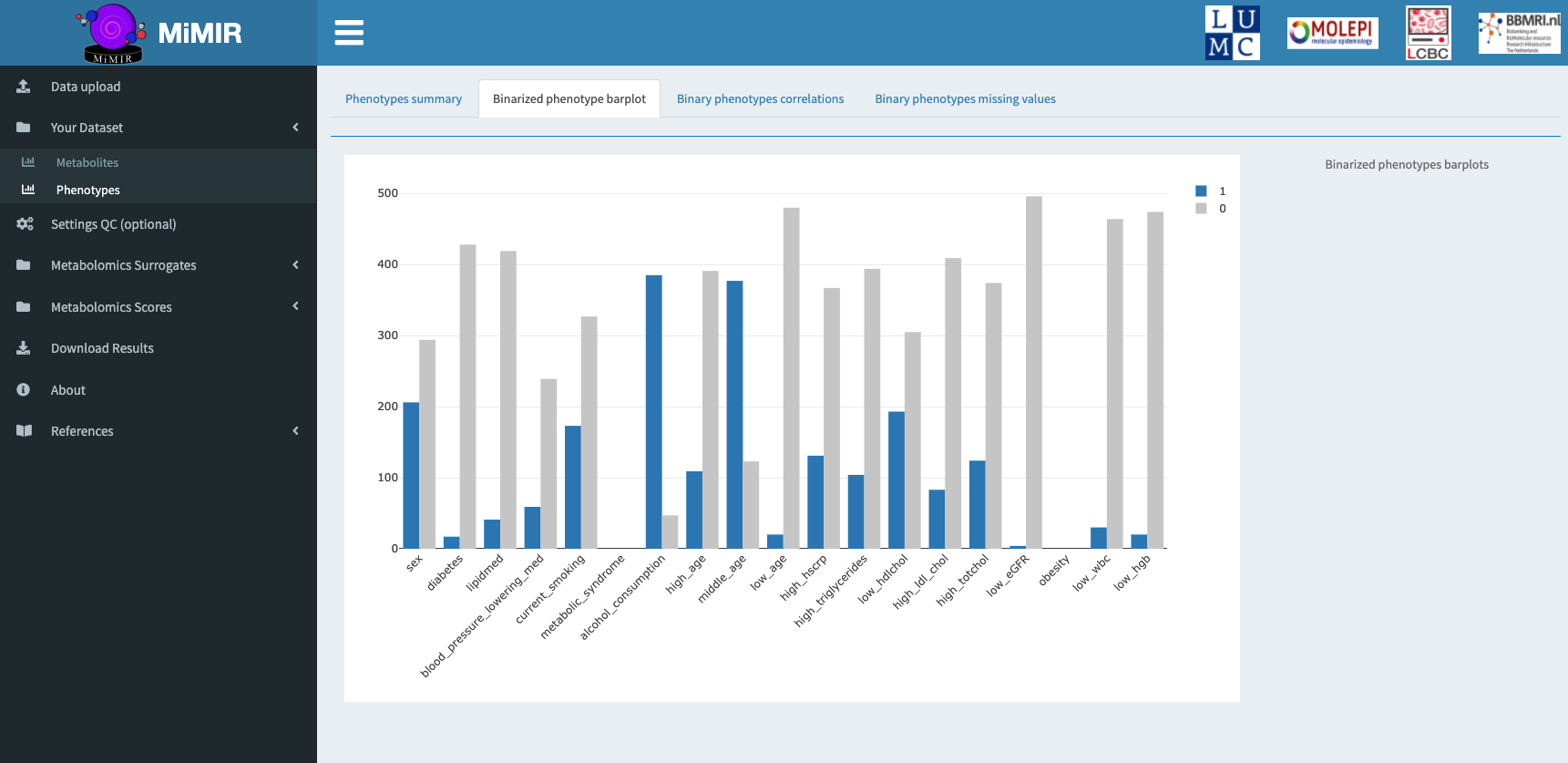


***Figure S11:*** The abundance of the binarized clinical variables used for the surrogate variables. For instance, this dataset is enriched with middle-aged individuals, alcohol consumers, relatively healthy, which don’t use medication.

1. Binary phenotypes Correlation: a heatmap representing the correlations between the binarized phenotypic variables. High correlations are indicated in red and low correlations in blue, as indicated in the legend on the right.


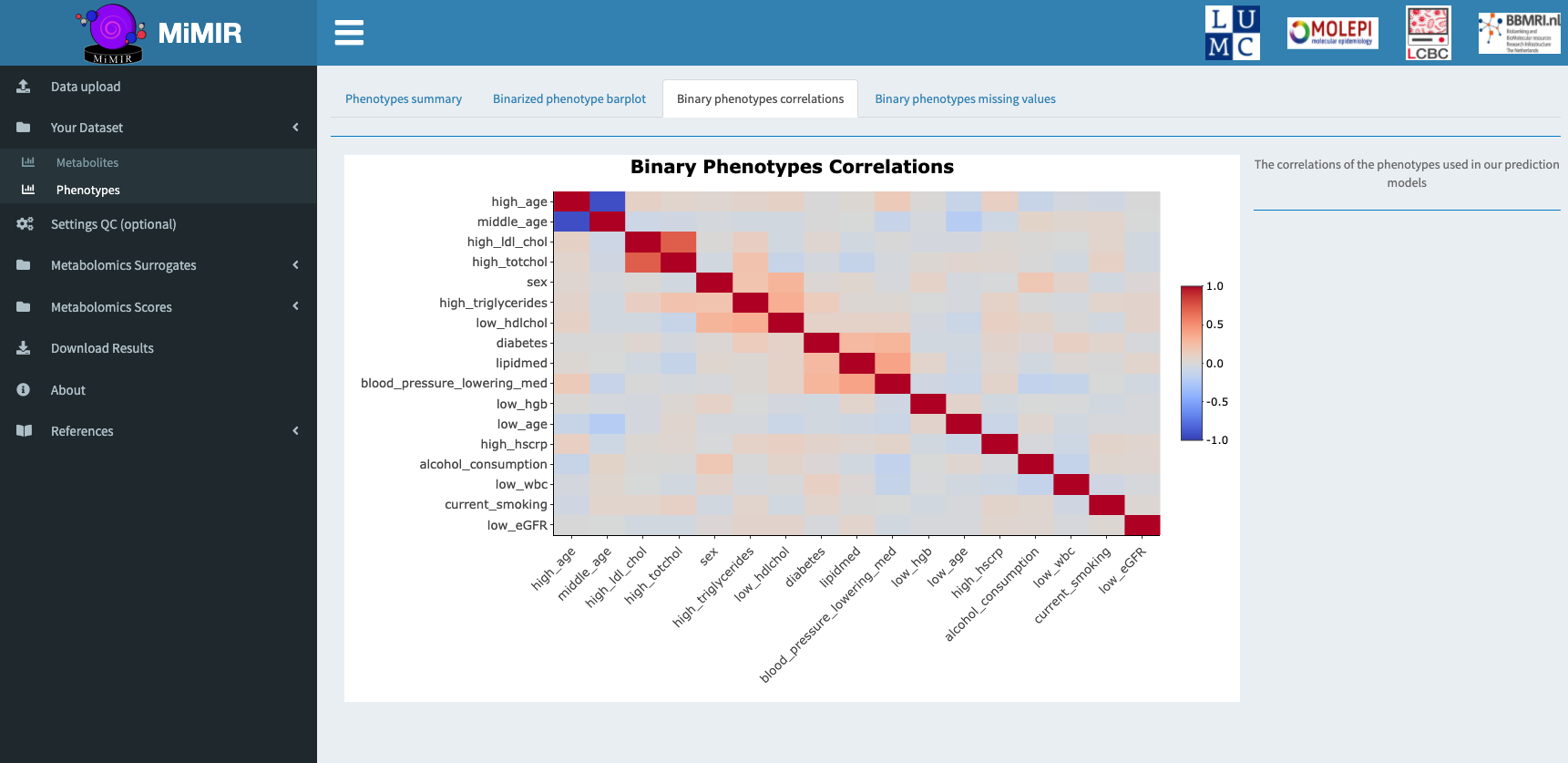


**Figure S12:** Correlation of the binarized clinical variables used for the surrogates. The correlation between these variables is generally very low.

1. Binary phenotypes missing values: It is a composed image representing the missing values in the binarized phenotypes. Similar to the missingness plot for metabolite values, this tab contains a heatmap with available values in grey and missing in white, and it also includes two bar plots on the sides: one to show the missingness per sample and another to present the missingness per phenotype.


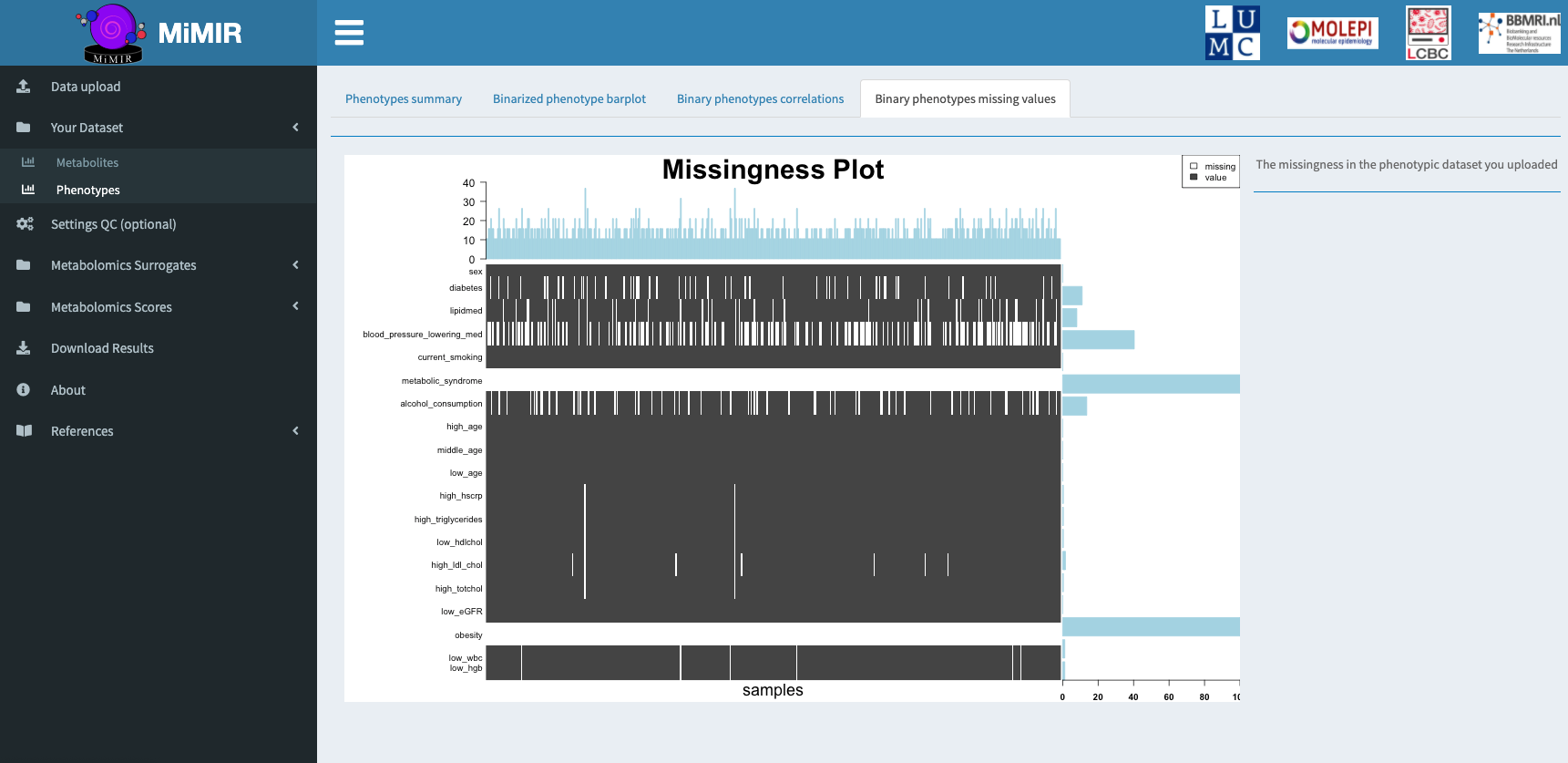


**Figure S13:** Missingness within the binarized clinical variables. There are not many missing clinical variables in LLS-PO, apart metabolic syndrome and obesity, which are completely missing.

## Tab 3: Settings QC (optional)

The Quality Control (QC) of the metabolic measurements is an important process which for the most part is already fixed by how the authors of the metabolic scores trained their corresponding models. This process generally consists in scaling the metabolic features, to select samples that were correctly measured and eventually impute the missing values. We included this tab to make the user able to have freedom, where possible, over the Quality Control Steps. Therefore, changing the settings in this tab is only optional and not necessary.

### Pre-processing MetaboAge

The pre-processing necessary to calculate MetaboAge on new cohorts consists of z-scaling the metabolites and imputing the missing values (with zeros). It is also necessary to filter samples with metabolic concentrations 5 standard deviations away from the mean, that have more than 1 missing value (Nmax_miss) or 1 zero (Nmax_zero). These last two values are the recommended values but could be changed in case of need using MiMIR. For instance, it could be possible to increase the number of allowed missing values per sample in cohorts in which some features were not possible to detect by Nightingale health, but in which the user still would like to obtain the values for MetaboAge.

### Preprocessing Metabolic surrogates

This preprocessing is very similar to the preprocessing of MetaboAge; however, the metabolic means and standard deviations are slightly different. Therefore, we allow to modify the QC thresholds separately.

### Pre-processing other scores

The other scores (mortality score, T2D score and CVD score) have a fixed quality control, so the QC Settings are not available for them. Here is a list of all the pre-processing steps for each score

- **Mortality score:** the metabolites are log transformed metabolites and then z-scaled. To avoid Infinite values after log transform it adds a +1 to the metabolites with zeros. It doesn’t involve any imputation. Therefore, an eventual missing value in the 14 metabolites that compose the score will also cause the mortality score to be unknown.
- **T2D score:** the metabolic concentrations are increased with +1, then log transformed and z-scale. It doesn’t involve any imputation.
- **CVD score:** it uses the same pre-processing as the mortality score.
- **COVID score:** metabolites outside 4 interquartile ranges from the median are considered as outliers and exclude. The remaining metabolites are z-scaled and used to calculate the score.

## Predicted Scores

After the user uploads the dataset, and optionally set up the Quality Control, MiMIR will calculate all the metabolic scores in parallel. The scores are divided in the surrogate scores, containing all the scores made by Bizzarri et al. [3] and the other scores, containing the rest of the calculated scores [1], [2], [5], [6]. This separation is due to the fact that it is possible to calibrate and calculate the AUC only for the surrogate values.

### Tab 4: Metabolic Surrogates

#### Metabolic Surrogates table

It shows 2 tables: the one including all the values for the surrogates and their calibrated values. In these tables the user is able to see each metabolic score results for each sample uploaded. The tables present 5 entries as default, but this can be changed to the user’s preference. It will also be possible to look for specific entries with the “Search” bar.


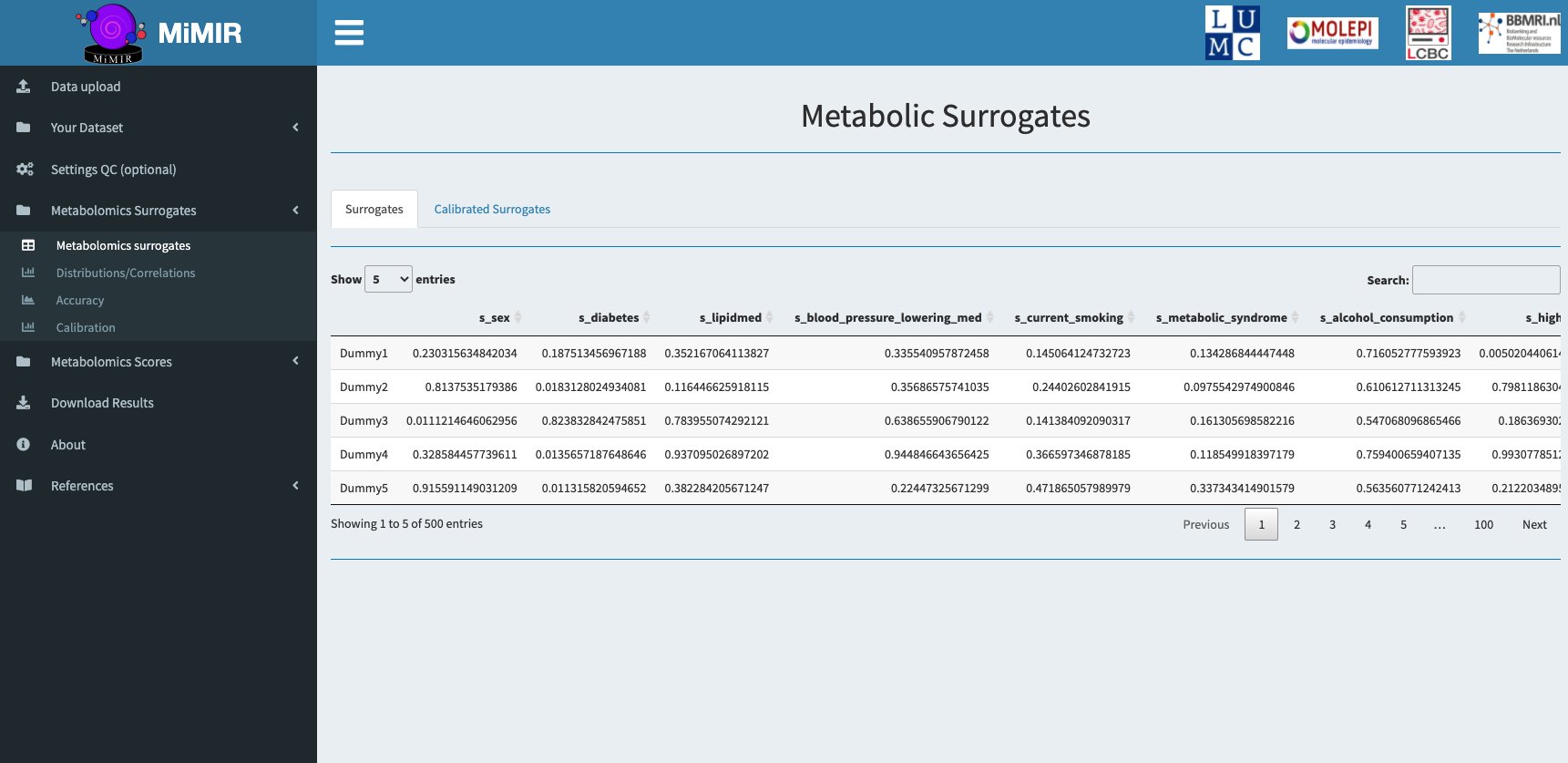


**Figure S14:** Values of the surrogates clinical variables in the first 5 samples of the synthetic dataset.

#### Distributions/Correlations

Also, in this case MiMIR will generate interactive plots for a quick analysis of the surrogate values:

1. Surrogates correlations: Pearson correlation between each pair of metabolic surrogate values.


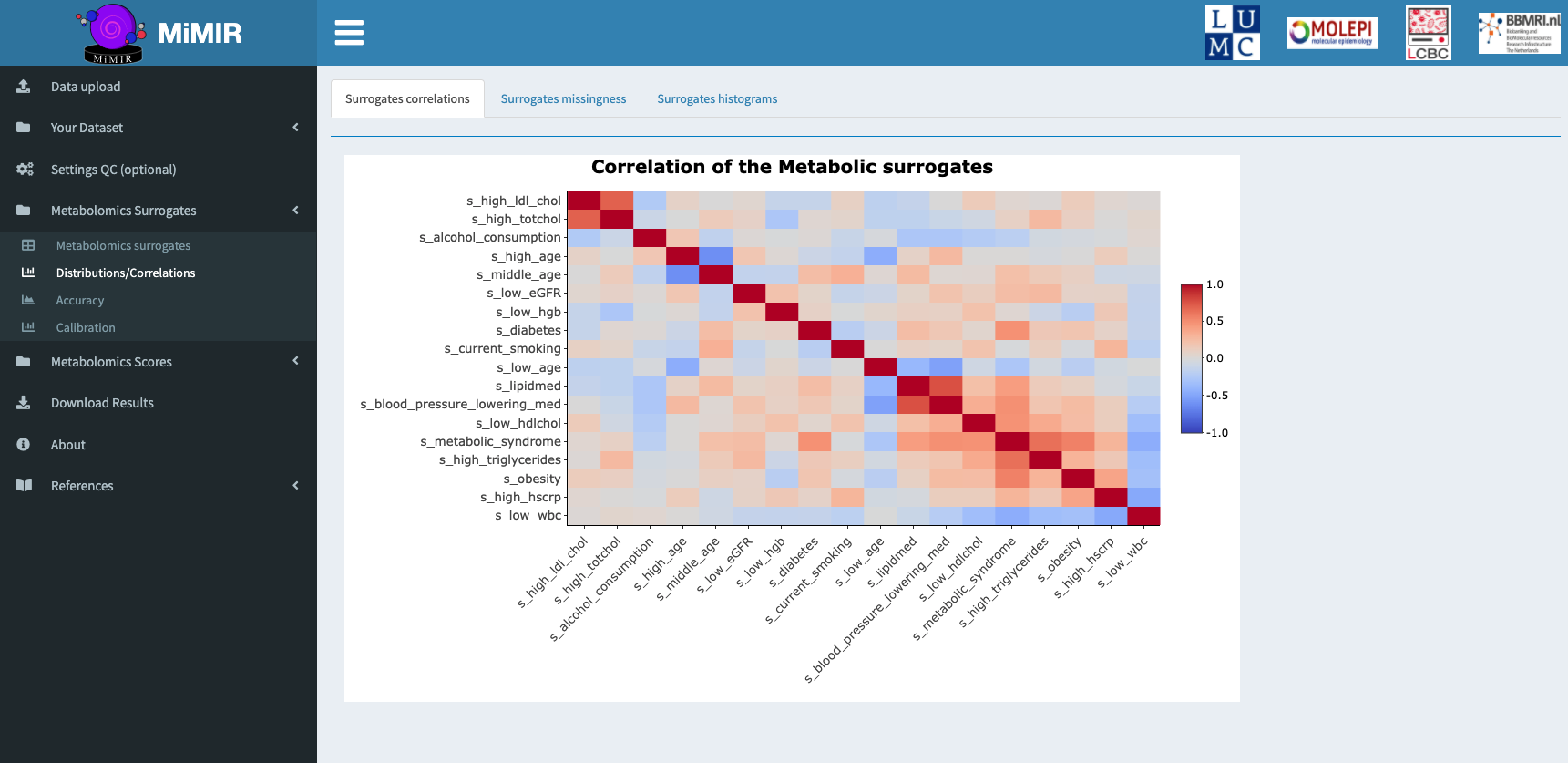


**Figure S15:** Pearson correlation between the surrogate variables. As shown in the original paper [], the surrogate variables show even some high correlation, but they seem to be driven by the nature of the clinical variables included (e.g. correlation between “blood pressure lowering med” and “lipidmed”) not only by the fact that they are scores made with only 56 variables.

1. Missing values: It is a composed image representing the missing values within the metabolic scores like the ones in the Metabolites and phenotypes tab. In this case, there should not be any missingness, because the surrogates were obtained using the metabolites, which were imputed in case of missing datapoints. Indeed, this plot, can be used as a check-up to control that the computations were correct.


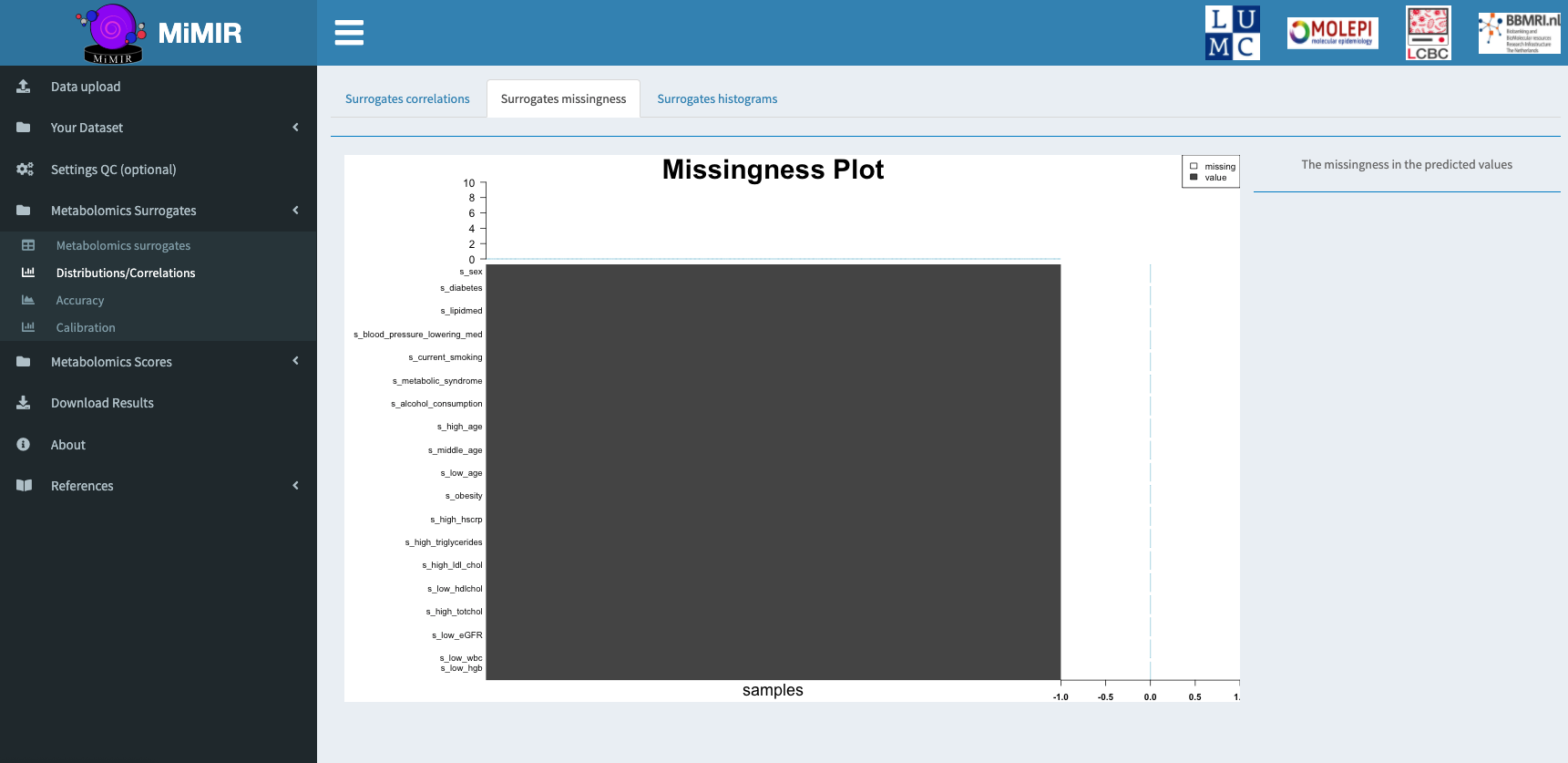


**Figure S16:** Missing observations in the surrogate variables. This plot should be always completely grey because the surrogates were calculated with all the available metabolites.

1. Surrogates histograms: this interactive bar plot shows the 19 metabolic surrogates distributions in the uploaded dataset. It is possible to load the distribution of one or multiple variables using the checkbox on the right side. Using a Radio Button, the user can also visualize their z-scaled version, to allow for comparisons.


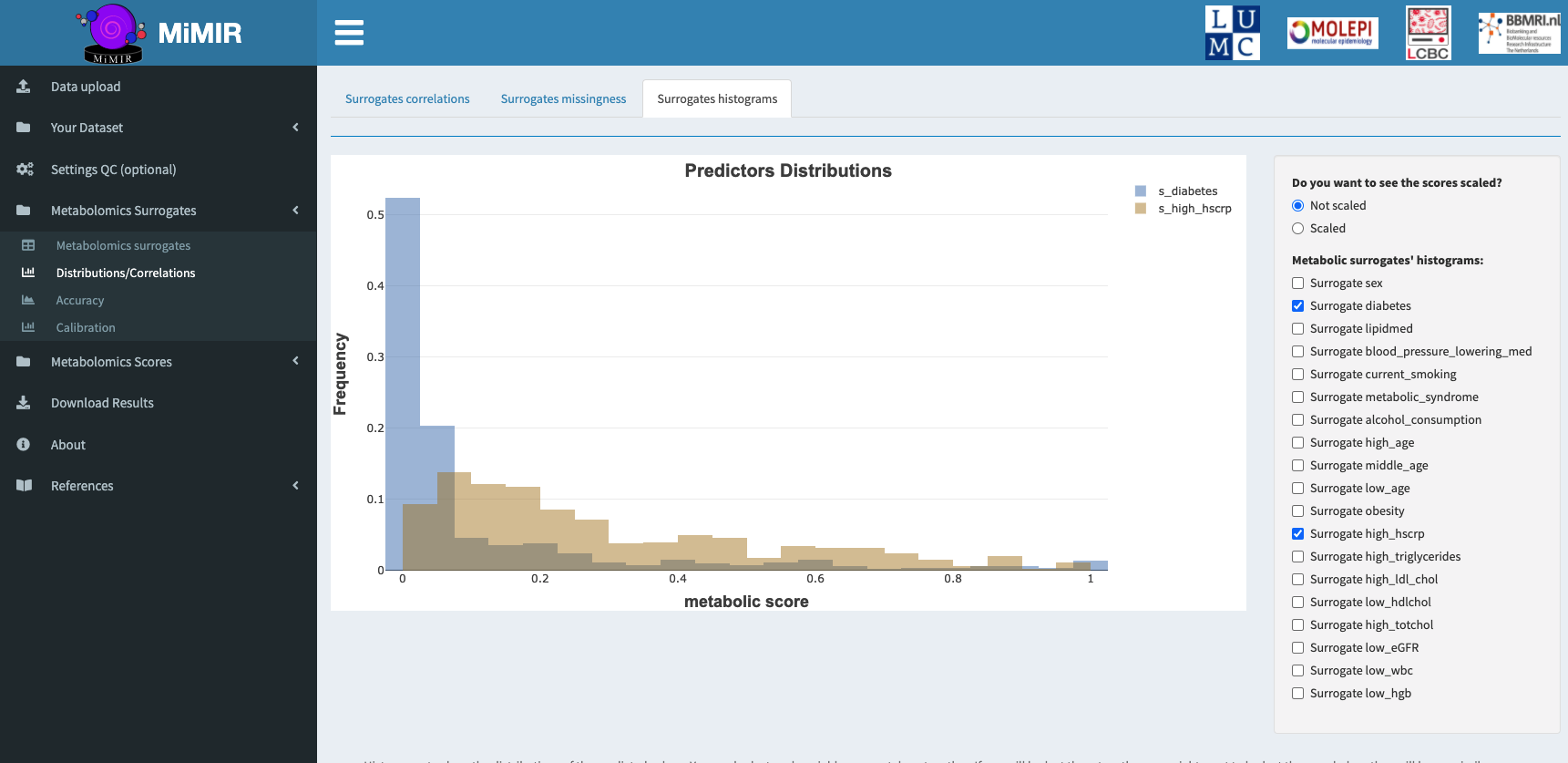


**Figure S17:** Distributions of surrogate ‘diabetes’ and ‘high hscrp’. While the subjects in LLS-PO have a low chance to be diabetics, they seem to have hsCRP levels rather high.

#### Accuracy

An important feature of MiMIR is that it allows to calculate the accuracy of the pre-trained metabolic scores within the uploaded dataset. To allow for the evaluation MiMIR will generate the following interactive plots:

1. Surrogates ROC curves: Receiver Operating Characteristic curve is a graphical tool that illustrates the accuracy of a binary classifier as its discrimination threshold varies. It is created by plotting the Sensitivity (True Positive Rate=TPR) against the Specificity (False Positive Rate=1- FPR) at various thresholds. Classifiers with a curve closer to the top-left corner indicate a better performance, while a curve closer to the 45$^{\circ}$ diagonal indicates worse performance. Using MiMIR it is possible to visualize the ROC for all the surrogate binary clinical variables in the uploaded dataset. It is also possible to evaluate multiple classifiers together, by selecting multiple surrogates from the checkbox on the side. The Area under the ROC Curve (AUC) is an aggregate measure of performance across all possible classifier thresholds and therefore it’s often used to evaluate the performance of a classifier. MiMIR will plot on the side of the ROC curves also the AUC of the selected surrogate


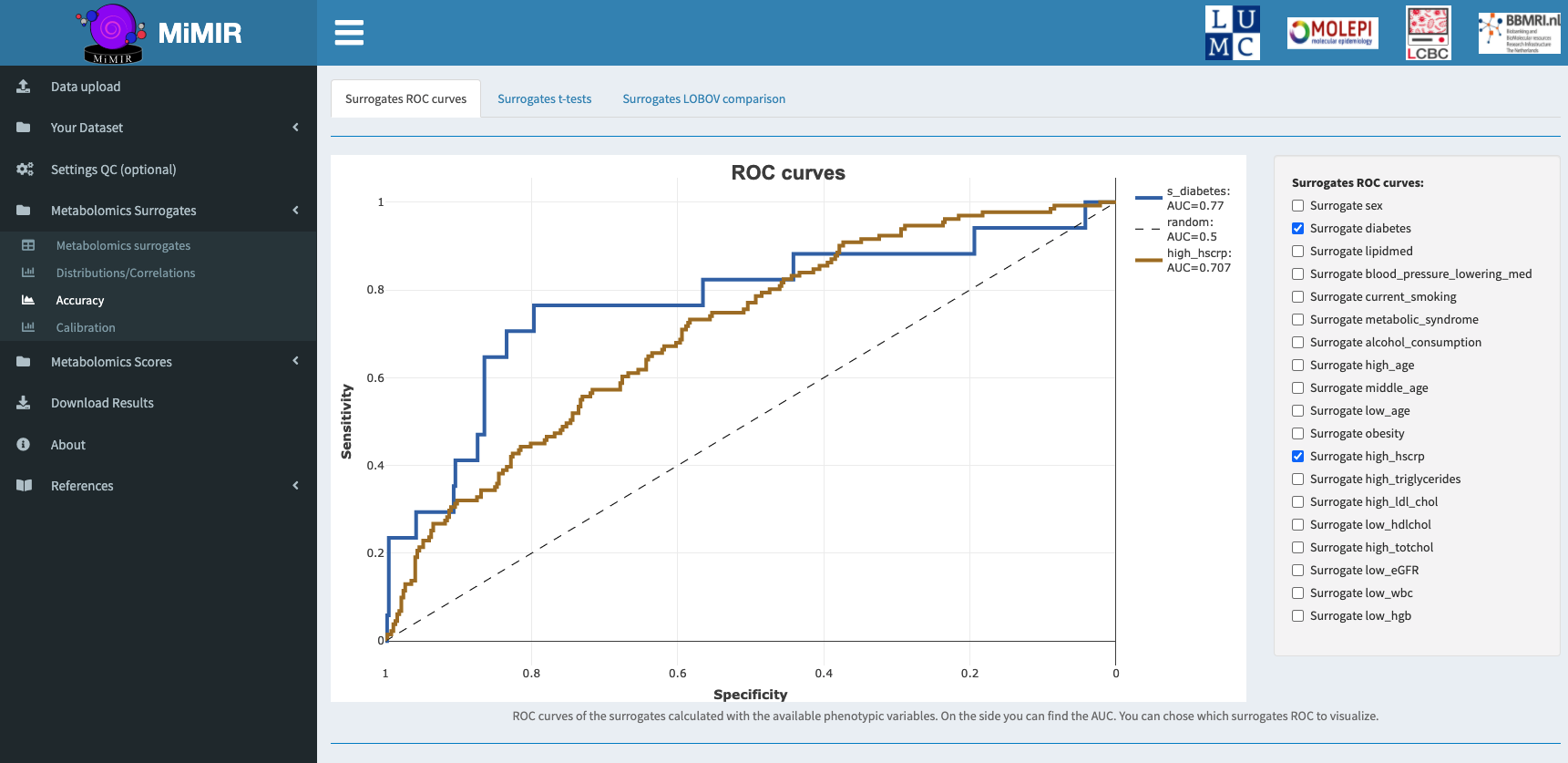


**Figure S18:** ROC curves of the surrogate ‘diabetes’ (blue, AUC=0.77) and ‘high hscrp’ (brown, AUC=0.707). These are the accuracy of the synthetic dataset, which are quite lower compared to what is obtained in the real LLS-PO dataset (AUC_diabetes_=0.85 and AUC_high hscrp_=0.812 in LLS-PO)

1. Surrogates t-tests: This Figure shows paired boxplots with the surrogate values split between the TRUE/FALSE (0 in blue, 1 in red) in the original values of the clinical variables. It will also produce t-tests to show how different these distributions are, and the resulting p-value will be show next to the surrogate names. In addition, this plot can be informative on the nature of the missingness of the phenotypic data (=NaN in grey). By looking at the distributions of the missing information it will be more or less evident if the subjects with missing information are at risk or not for certain clinical variables.


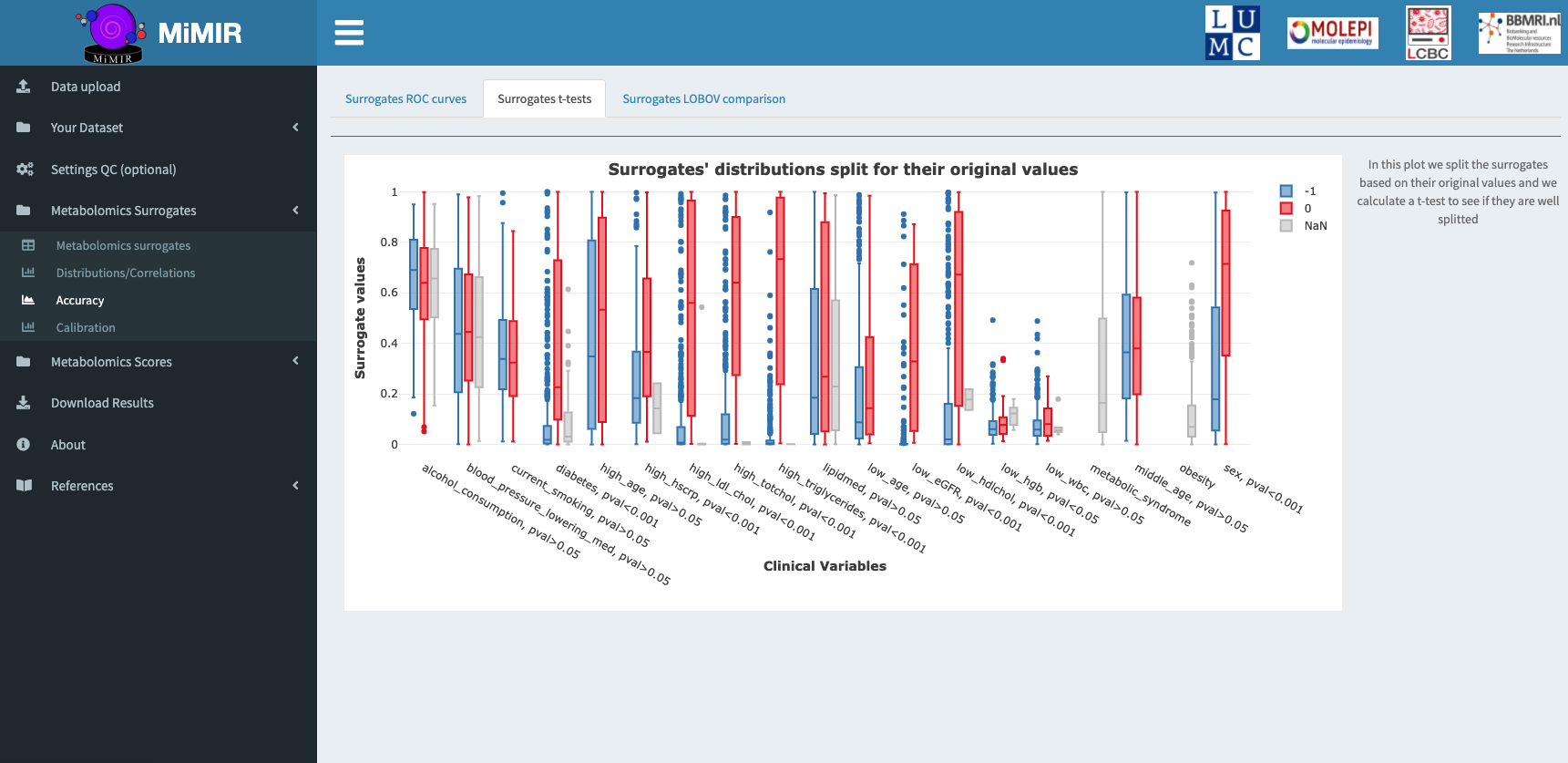


**Figure S19:** T-test indicating if the surrogate variables could split well the original clinical variables in LLS-PO. Generally, all the t-tests indicate that there is a good separation between TRUE/FALSE, however some variables have a clearer separation (e.g. ‘sex’) than others (e.g. ‘alcohol consumption’). Moreover, it is possible to visualize where the missing variables lie in the distributions of the surrogates.

1. Surrogates LOBOV comparison: In the paper of the surrogate clinical variables we applied two training-testing procedures to evaluate the models created. The first one is a Double 5Fold Cross Validation, which serves to fairly evaluate the model on the entire BBMRI-nl dataset. The second evaluation procedure consisted in a Leave One Biobank Out Validation (LOBOV), which consists of holding out one of the biobanks from the training set to be used as a test set. This evaluation technique was done to simulate the accuracies of the surrogate models on a biobank “external” to the training set. Therefore, we included the results of the LOBOV analysis in MiMIR to serve as a comparison for the accuracies achieved in the uploaded dataset (in blue) compared to the accuracies in the cohorts of BBMRI.nl (in red).


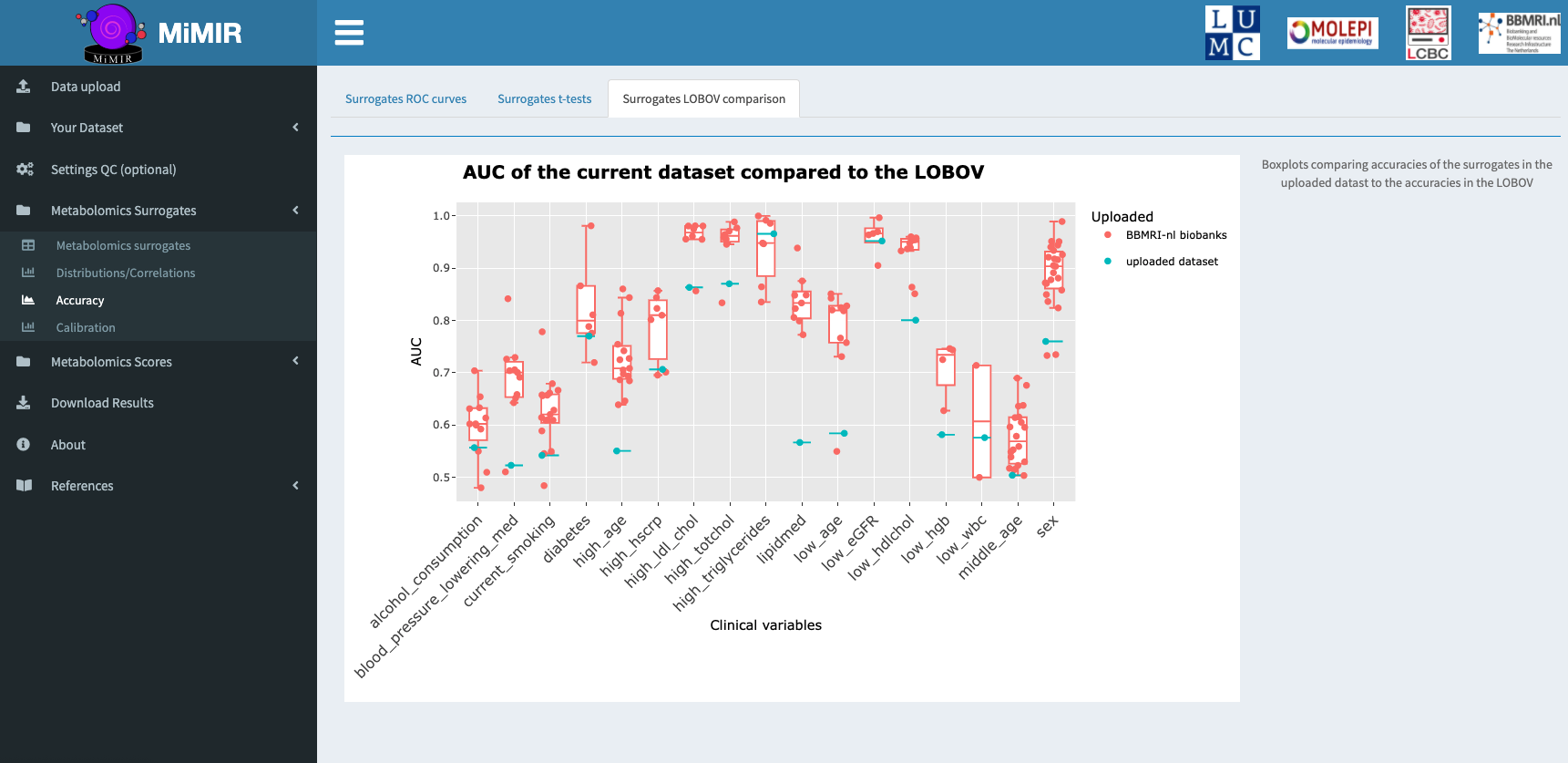


**Figure S20:** Comparison in the AUCs of the surrogates in LLS-PO and the results of the Leave One Biobank Out Validation. The performances in the synthetic dataset are generally all lower compared to all the other real dataset. Please look at Figure 1 in the main text to see the results in LLS-PO.

#### Calibration

We implemented the calibration only for the surrogate clinical variables, which were built as logistic regression models. Therefore, we focused on the class prediction and used one of the most popular methods: Platt scaling [19].

##### Platt scaling

Suppose m is a model which given an input X matrix can predict a class $\hat{y}$ with a probability $\hat{p}$: m(X)= $\left( \hat{y} \right| \hat{p})$. $\hat{p}$ is also called confidence and it represents the probability of correctness of the model, and for the model to be calibrated, we expect $\hat{p}$ to always represent the true probability p:

$$\mathbb{P}\left( \hat{y}=y \right| \hat{p}=p)=p$$

for all probabilities $p\in[0,1]$. Where the left-side represents the probability distribution given that the model predicts $\hat{p}=p$, while the right side represents what should be the calibrated value. Any difference between the right and left sides for a given p is known as calibration error. Well calibrated posterior probabilities can be achieved through various model post-processing strategies like re-calibration. Re-calibration describes the mapping of a raw predictor output to the probability domain [0;1]. A popular calibration algorithm is the one defined as Platt scaling. John Platt created the following parametric method to transform the output of a Support Vector Machine into posterior probabilities. However, any classifier’s output can be post-processed with Platt Scaling [19]. Platt’s idea consists in passing the row model estimates through a logistic regression model:

$$\mathbb{P}\left( \hat{y}=1 \right| \hat{p})= \frac{1}{1+e^{A\hat{p}+B}}$$

Where $\hat{p}$ denotes the output of the model’s prediction and the A and B are fit using maximum likelihood estimation from a training set. MiMIR uses a straightforward implementation of Platt scaling in prost-processing using the Logistic regression implemented as the *glm* function of base R [20]. The logistic regression (LR) maps the raw predicted probabilities to true binary outcome. To evaluate this calibration and avoid overfitting, we train the LR on the 80% of the uploaded dataset and evaluate it on the remaining 20%.

##### Reliability diagrams

The reliability diagrams (Figure S22) are visual inspection tools for model calibrations [21]–[23]. These diagrams present the sample accuracy as a function of the confidence. To estimate the expected accuracy, we order the prediction values and group them into J equally spaced intervals (each of since 1/J), normally in 10 bins, like in Figure S21


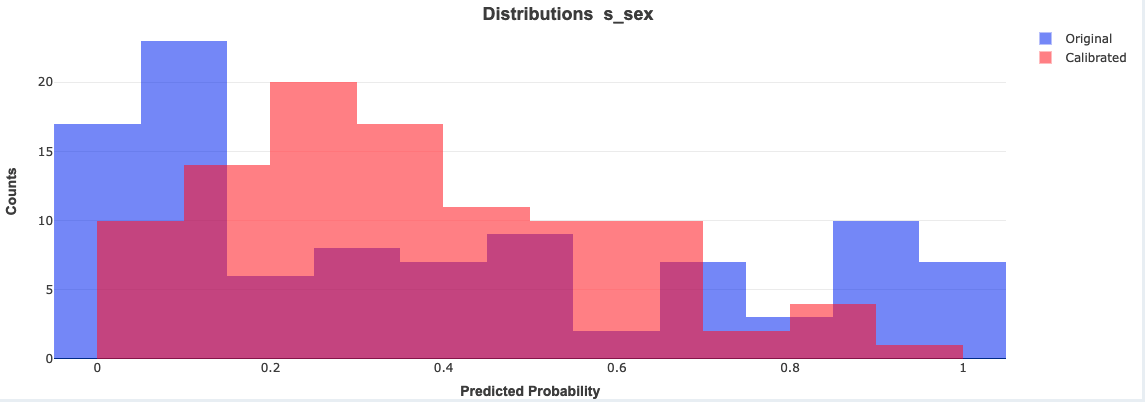


**Figure S21:** Distribution of the surrogate ‘*sex*’ (blue) and its calibrated version (red) split in 10 bins.

Each bin includes a set of samples whose prediction confidence are included in the interval $I_{j}$= $\left( \frac{j-1}{J},\frac{j}{J} \right]$. The accuracy of that bin is proportional to the number of predicted values $\hat{y}_{i}$ are equal to the true labels $y_{i}$, divided by the number of samples in that bin ($B_{j})$:

$$acc\left( B_{j} \right)=\frac{1}{{|B}_{j}|} \sum_{i\in B_{j}} 1 (\forall\hat{y}_{i}=y_{i})$$

The average confidence of that bin $B_{j}$ is defined as:

$$conf\left( B_{j} \right)=\frac{1}{{|B}_{j}|} \sum_{i\in B_{j}} \hat{p}_{i}$$

where $\hat{p}_{i}$ is the confidence for the sample *i*. In practice a reliability diagram plots the mean predicted value against the fraction of accurately predicted values. A perfectly calibrated model will have $conf\left( B_{j} \right)=acc\left( B_{j} \right)$ for all the bin intervals N. So, if the model is well calibrated the points will fall near the diagonal line. MiMIR allows to produce reliability plots for all the metabolic surrogates and it allows to interactively set the number of bins J.


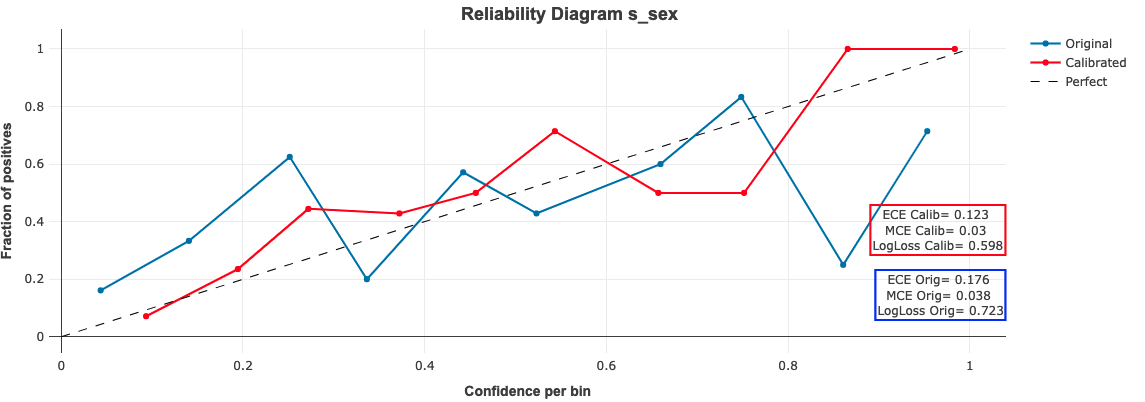


**Figure S22:** Reliability Diagram with ‘*sex*’ (blue) and its calibrated version (red). We can notice how the calibrated ‘*sex*’ is closer to the diagonal, meaning that the calibration worked on this specific dataset. We can also evaluate the improvement looking at the decreased value of ECE, MCE and LogLoss (ECE_calib_ -ECE_orig_= -0.053, MCE_calib_ – MCE_orig_=-0.08, LogLoss_calib_-LogLoss_orig_=-0.125).

##### Calibration performance measures

**Calibration errors:** While reliability diagrams are useful visual representations, it is convenient to have a summary statistic of the calibration’s performance. One of the most used measures is the **Expected Calibration Error (or ECE)** [ref], which measures the difference in expectations between confidence and accuracy, i.e.:

$$\mathbb{E[|P}\left( \hat{y}=y \right| \hat{p}=p)-p|]$$

To approximately measure the calibration error, it is common use to discretizes the probability interval into a fixed number of bins, like the Reliability Diagrams:

$$ECE=\sum_{j=1}^{J} \frac{{|B}_{j}|}{n}\left| \begin{aligned} \\ acc\left( B_{j} \right)- conf\left( B_{j} \right) \end{aligned} \right|$$

where n is the number of samples. At the same time MiMIR reports also the **Maximum Calibration Error (MCE)**, which is normally used to evaluates the maximum difference between accuracy and confidence [23], [24].

$$\max_{p\in[0,1]} \mathbb{|P}\left( \hat{y}=y \right| \hat{p}=p)-p|$$

Which approximated becomes:

$$MCE=\max_{j\in\{1,\ldots,K\}} \left| \begin{aligned} \\ acc\left( B_{j} \right)- conf\left( B_{j} \right) \end{aligned} \right|$$

MiMIR reports the ECE and MCE of both the calibrated and non-calibrated measures to let the user be able to compare them and decide if the Platt scaling positively impacted the posterior probability. If the ECE and MCE are lowered, it means that the calibration was successful.

**Log-loss:** The Log-loss (LL) is another measure used to assess the quality of predictor models probability. It is referred to also as “negative log likelihood” or “cross entropy loss”.

Which for binary classification can be written as:

$$logloss=- \frac{1}{n}(y log \hat{p})+(1-y)log(1-\hat{p})$$

where $\hat{y}_{i}$ is the true binary outcome and $\hat{p}$ is the predicted probability that the observation belongs to that class [23]. Log-loss increases as the predicted probability diverges from the actual label. A perfect model would have a log loss of 0. MiMIR reports also in this case this measure for the calibrated and the original surrogates, so the user can compare the two values.

### Tab 5: Metabolomics Scores

#### Metabolic scores tables

In this section it’s possible to inspect the values of each metabolic score calculated by MiMIR separately or together (comprising also the surrogate scores).

In case MiMIR is not able to calculate on of the scores it will describe the error to the user.

If a “variable x” wasn’t recognized in the inputs the error will look like:

| It was not possible to calculate the score because of the missing variable(s): “variable x” |
| --- |

In case the values of a “variable x” are completely missing then the error will be:

| It was not possible to calculate the score because all the values of “variable x” are missing |
| --- |

#### Distributions/ Correlations

In this section the user can find interactive plots to study of all the metabolomics-based scores.

1. Predictors correlations: it visualizes the correlation between each pair of metabolic scores, including also the surrogate variables.


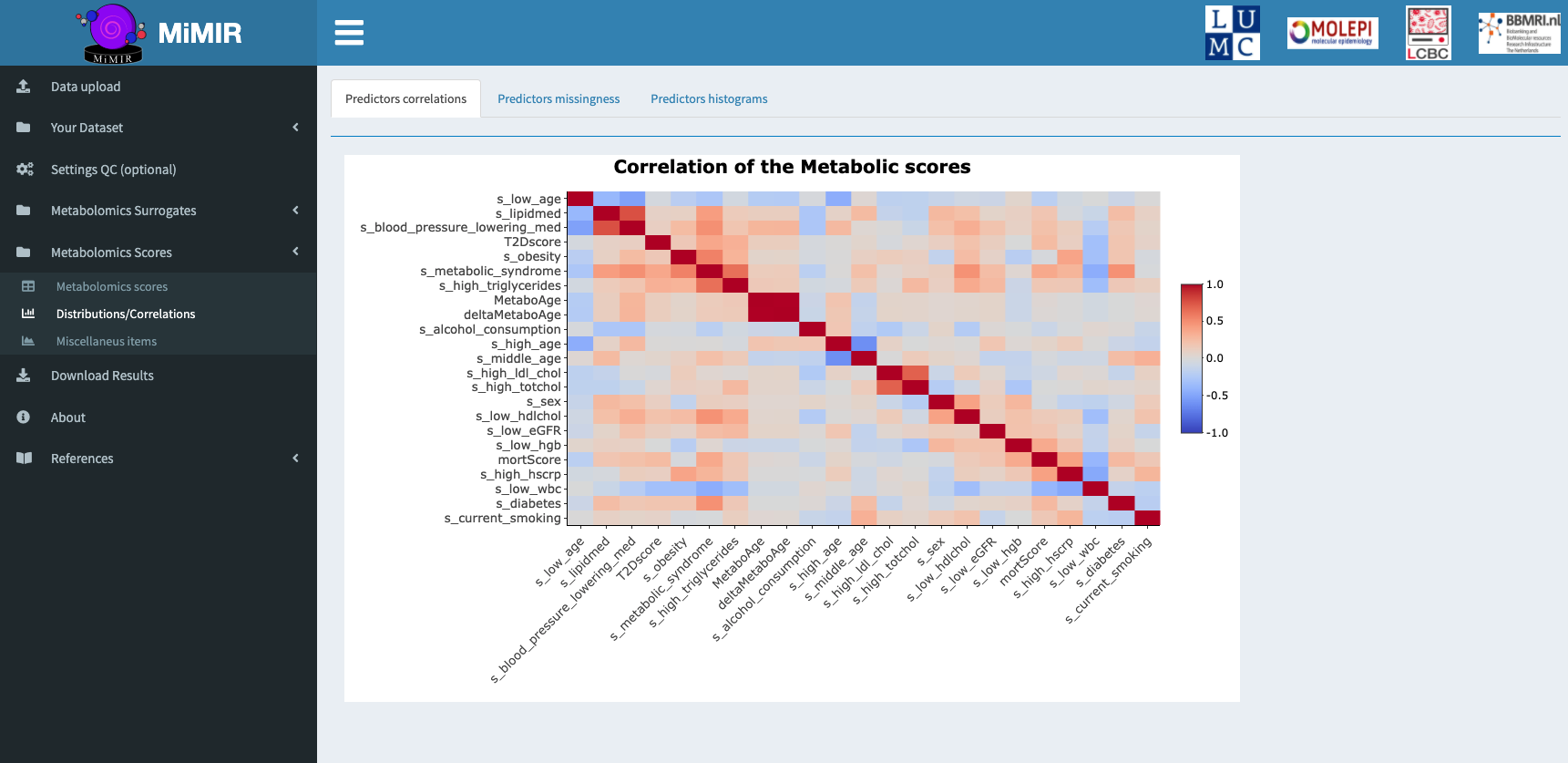


**Figure S23:** Correlation of all the metabolomics-based scores.

1. Predictors missingness: It is a composed image representing the missing values within the metabolic scores like the ones in the Metabolites and phenotypes tab. In this plot you can see the missingness in the available scores. Some values might be missing because of the missingness in the used metabolites or variables.


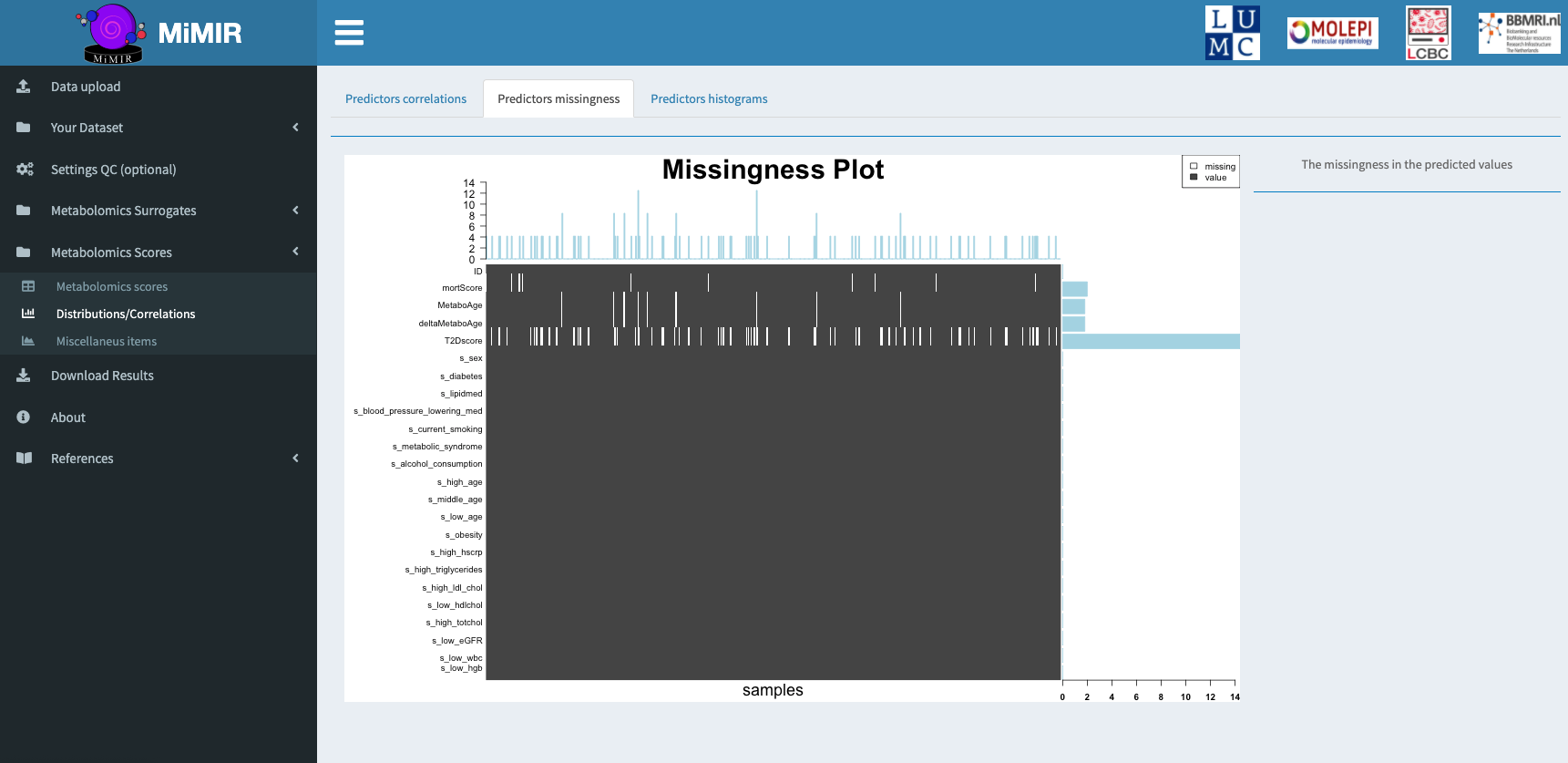


**Figure S24:** Missingness plot of all the metabolomics-based scores. Here, we can see different patterns in missing observations, due to the different pre-processing steps. For instance, the scores that don’t include the imputation step will have more missingness (e.g., the mortality score).

1. Predictors histograms: this interactive bar plot shows the distributions of all the 24 metabolic scores in the uploaded dataset. It is possible to load the distribution of 1 or multiple variables. Using the radio button, on the top right, the user can also visualize their z-scaled version, to allow for comparisons.


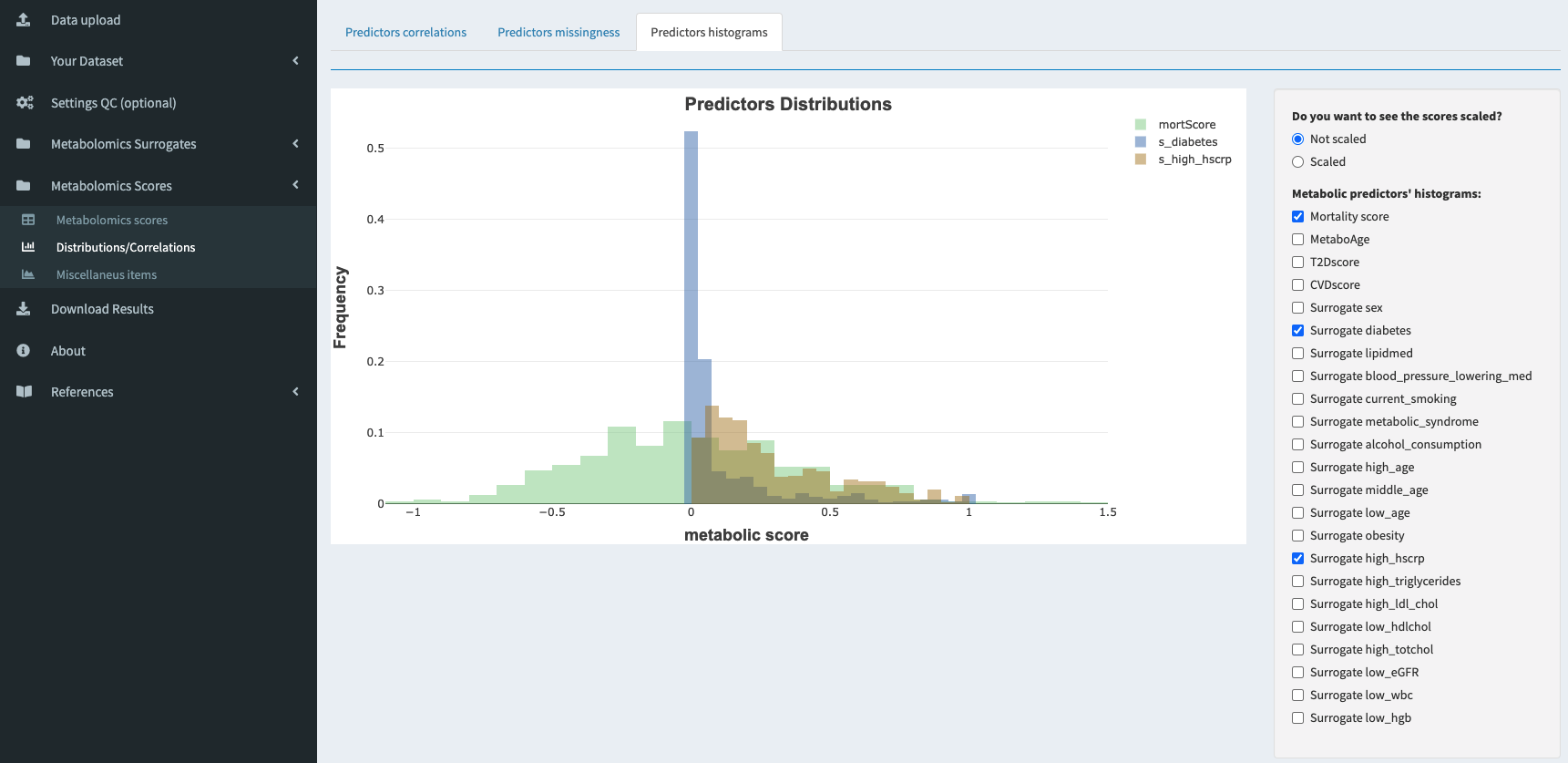


**Figure S25:** Distributions of the ‘*mortality score*’, ‘*diabetes*’ and ‘*high hscrp*’. Some of these scores have a totally different distributions, so it might be necessary to scale them using the radio button on the right to compare them.

#### Miscellaneous items

This section allows to evaluate the accuracy and other analyses using the metabolic scores:

1. MetaboAge/age accuracy estimation: This scatterplot presents on the x-axis the chronological age of the individuals (uploaded in the phenotypes file) and on the y-axis MetaboAge. To accompany this visual inspection tool MiMIR also calculates some accuracy measures useful to evaluate continuous variable predictions: *R*, $R^{2}$ and finally the median error. *R* is the Pearson correlation coefficient calculated between MetaboAge and chronological age. This value is useful to understand if MetaboAge is mimicking well the chronological age, but it’s strongly dependent on the standard deviation of age. The median error instead is used to study if the predictor is poorly calibrated. To have a clear evaluation we also plot a regression line in red and a dotted line representing y=x. In case of a perfect estimation these two lines would be equal. However, linear regression is often affected by the regression to the mean problem, which consists in giving prediction more toward the mean of the original value to avoid making incorrect inferences [25].


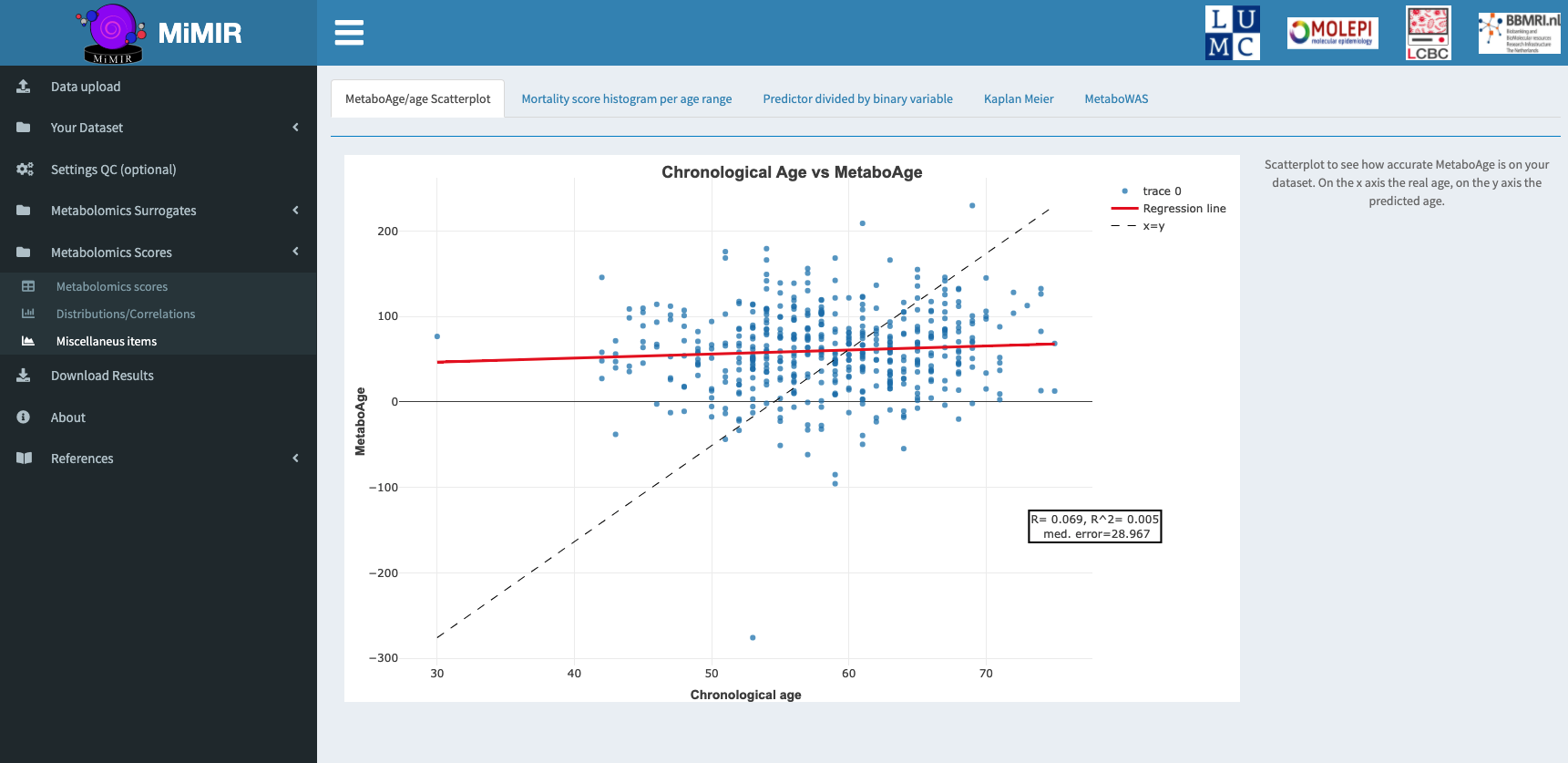


**Figure S26:** Scatterplot comparing chronological age to MetaboAge. The performances of MetaboAge was extremely affected by the use of the synthetic dataset, as it was for all metabolomics-based age predictors (high age, middle age and low age)

1. Mortality score histogram divided in age ranges: This consists of a histogram showing the mortality scores distribution divided in 3 different age ranges: age<45 y.o., 45 y.o. $\geq$age<65 y.o. and finally age $\geq$ 65 y.o. Since we consider the mortality score as an all-around biomarker of health the score should be higher for higher ages.


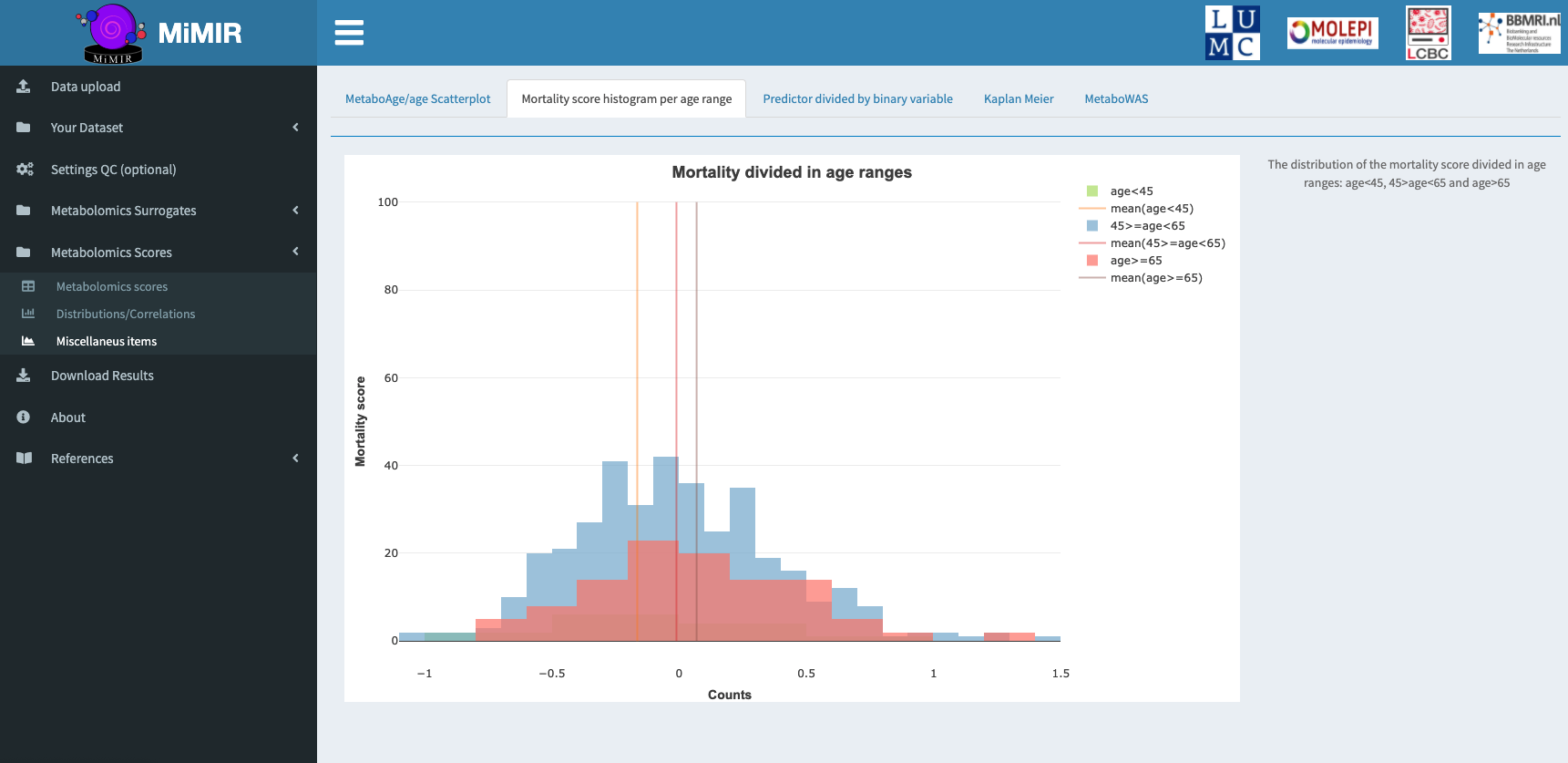


**Figure S27:** The distribution of the mortality score divided in three age ranges: lower than 45 years old (green), between 45 and 65 years old (blue) and higher than 65 years old (red). We can see how the higher the age is the higher the median of the mortality scores increases.

1. Metabolomics-based score divided by binary variable: an interactive bar plot reporting the values of one of the available metabolic scores (on the y axis) split using a binary variable uploaded in the phenotypic file by the user. MiMIR will also compare the two groups of value performing a t-test and reporting the p-value.


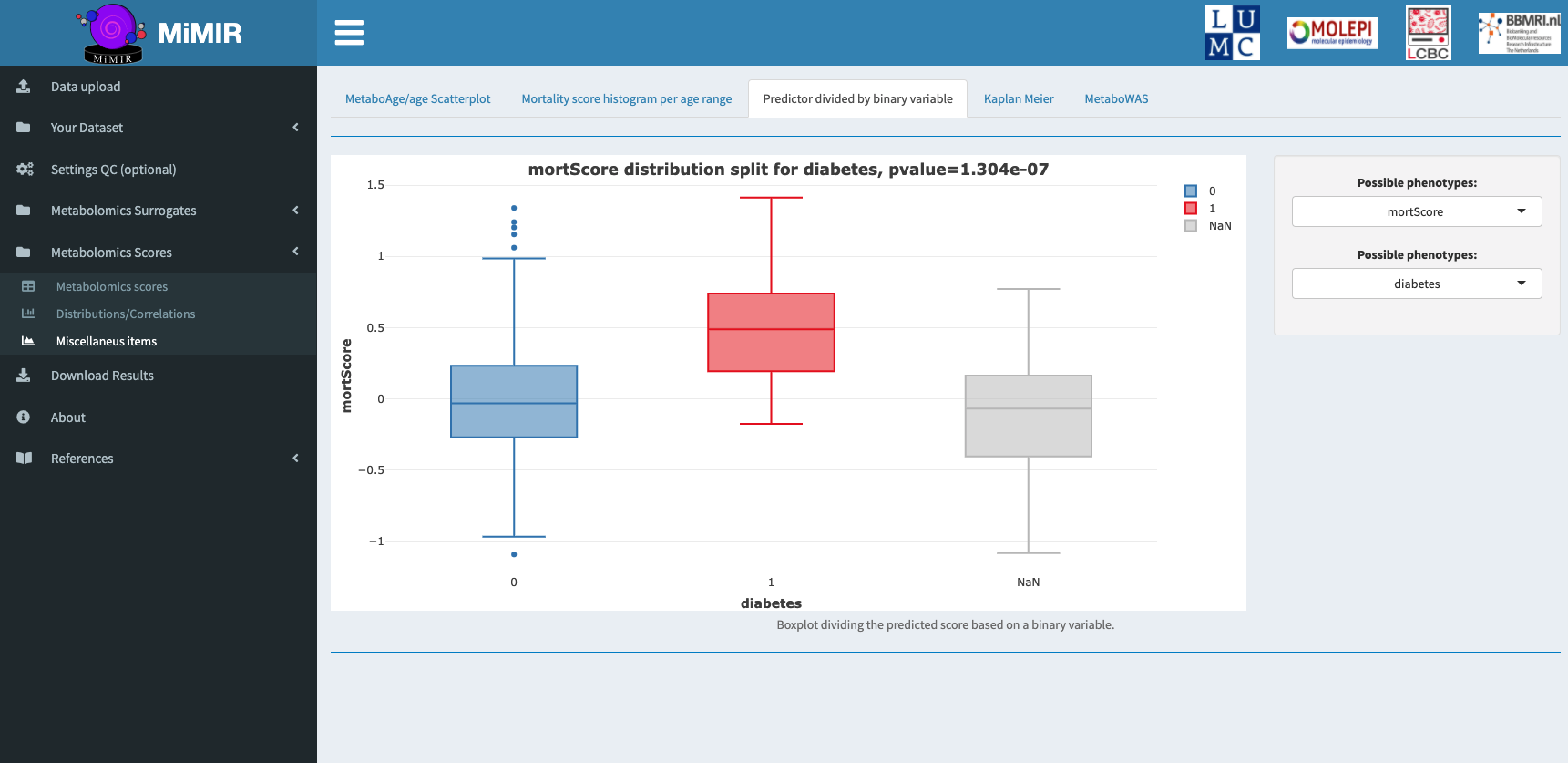


**Figure S28:** Comparison of the mortality score between diabetics and non-diabetics. The diabetics people have an increased mortality score with a highly significant difference (t-test p-value= 1.304 ${10}^{-07}$).

1. Kaplan Meier: The Kaplan-Meier curve is defined as the probability of surviving while dividing time in small intervals [26]. The Kaplan Meier curves permit to compare groups of people based on their survival. What this interactive image do is to compare the survival of the people in the 1^st^ and 3^rd^ tertiles of one of the scores available.

The event information is uploaded in the variables “Event” and “EventAge”, which are included in the phenotype file. The variable Event is the event indicator:

Event=1 if the event was observed (e.g. T_i_ $\leq$ C_i_)

Event=0 if censored (e.g T_i_ $>$ C_i_)

with T_i_ indicating the event time and C_i_ indicating the censoring time. And the variable EventAge indicates the age (in years) of the subjects at censoring time.

The user can test this association with any of the metabolomics-based scores available using the select box on the right and specify the name of the event by typing it in the text bar.


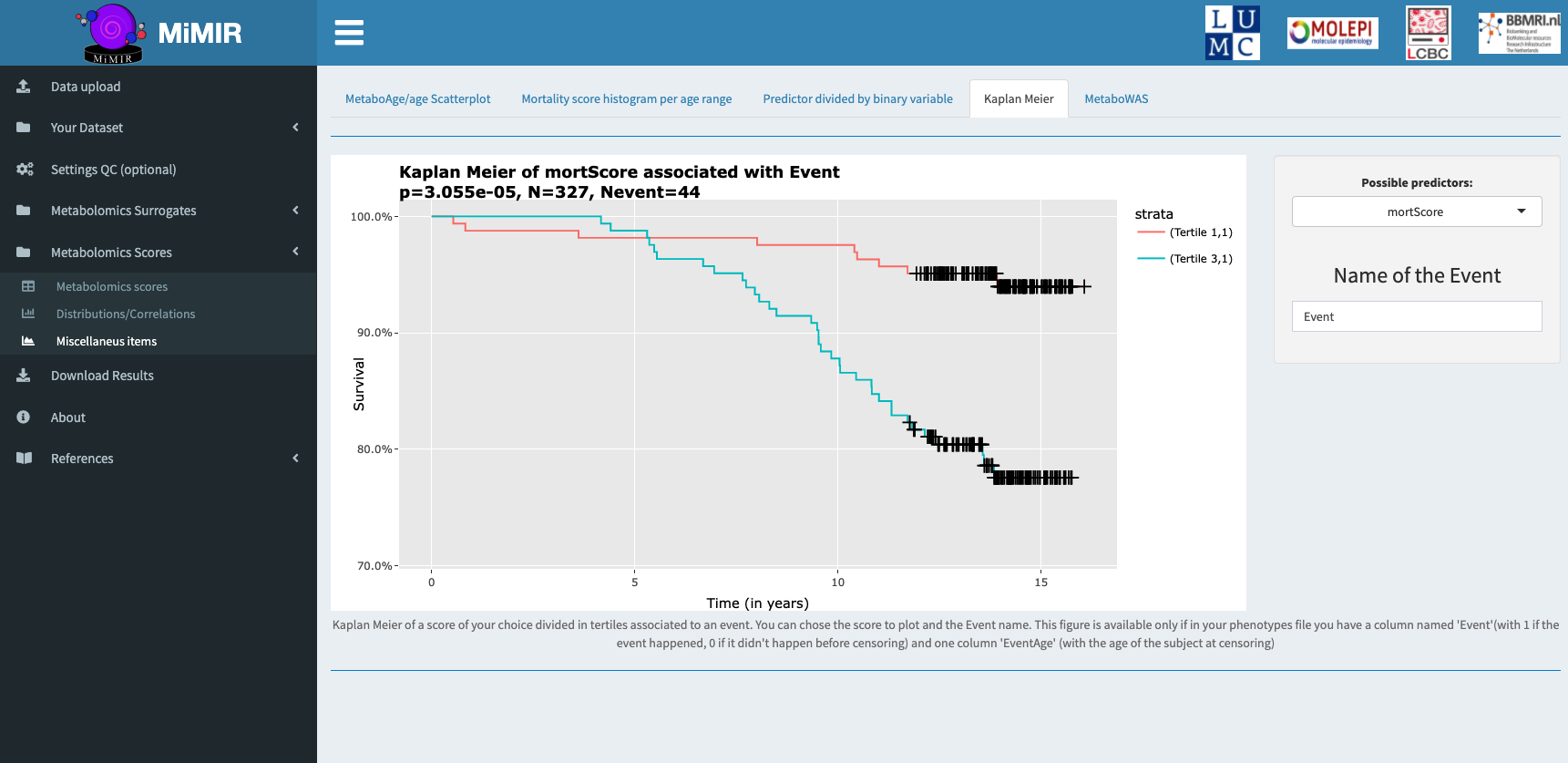


**Figure S29:** Kaplan Meier comparing the Time to Death of the participants divided according to the mortality score, where the first tertiles includes participants with a lower mortality score (red), while third tertiles the ones with a higher mortality score (blue). We can notice how people with a higher mortality score are more inclined to die faster, even in this synthetic dataset (Cox Regression p-value= 5.055 ${10}^{-05}$).

1. MetaboWAS: Metabolome Wide Association Study, which is an approach that study the association of each metabolite available in the Nightingale Health platform with a “test variable”. This is done by training linear regression models, adjusted by potential confounders. False Discovery Rate (FDR) is applied to account for multiple testing correction. The user has the faculty to select the test variable and the potential covariates within the pool of variables in the phenotypic file input and the metabolomics-based scores.

The results of the associations are reported in a Manhattan plot and a table with the summary statistics of the linear model for each metabolite. The interactive Manhattan plot reports on the x-axis the list of metabolites reported in the Nightingale Health, divided in groups, and on the y-axis the -log (adjusted p-value). Each dot represents a metabolite and passing over it with the mouse will reveal all the information.


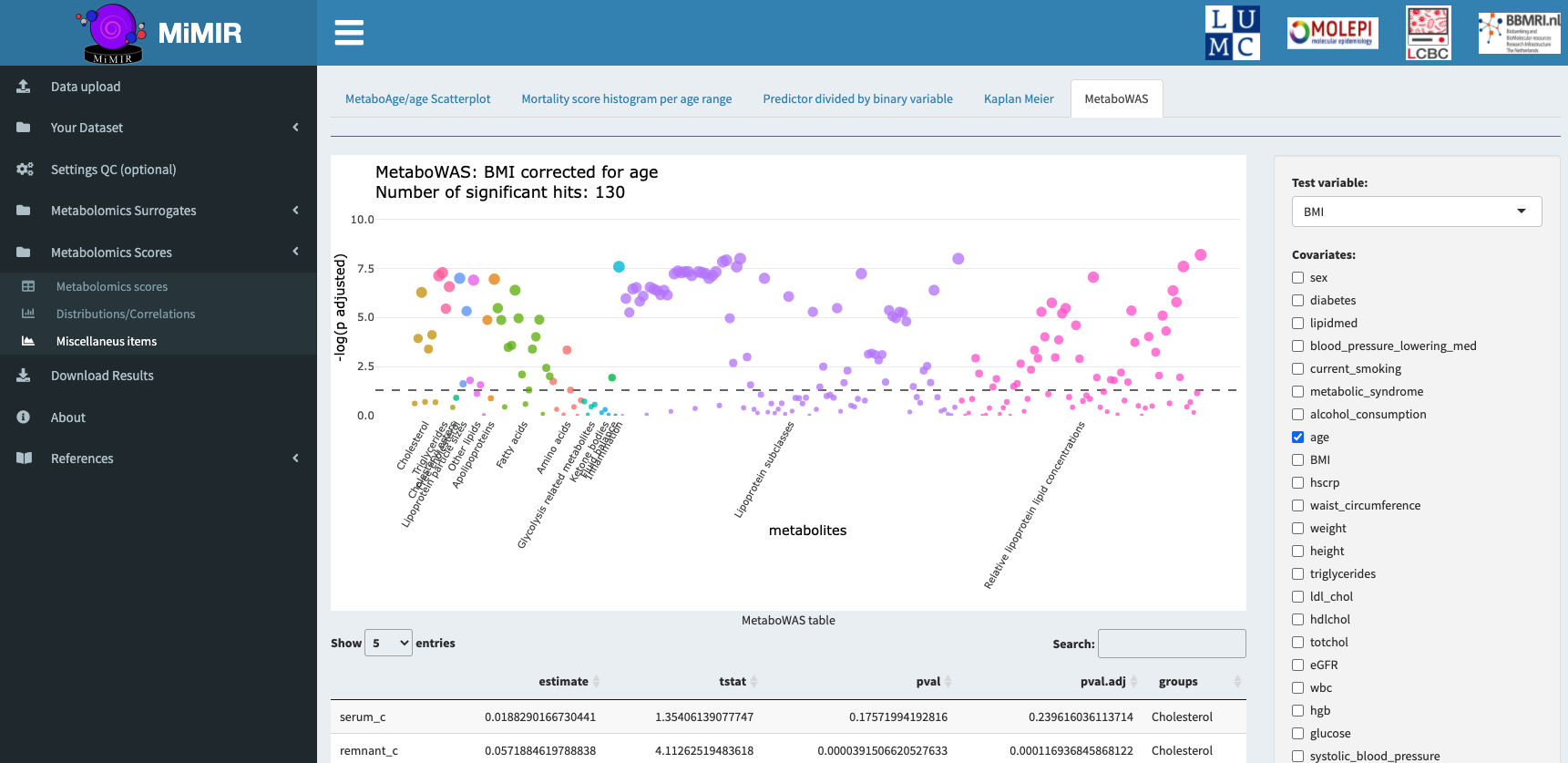


**Figure S30:** Results of a MetaboWAS for BMI corrected for age and sex. Not surprisingly, the most significantly associated metabolite is Glycoprotein acetyls, which is an inflammation marker often associated with BMI

.

## Download Results

#### Download a plot

Downloading most of the plots can be done directly from the R-shiny application. Most of the pictures in this app (all apart the missingness heatmaps) are obtained using the package Plotly [7], which allows to have interactive images. Moreover, going over the plots you will be able to see this taskbar in the top-right corner:


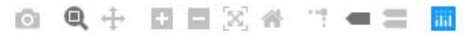


With this toolbar you can zoom in and out in the plot, zoom in a selection, move the axis, reset the axis, toggle spike lines to be able to better visualize some points and finally download the plot as a .png. To save the plotly images in high resolution images we suggest the package orca() (<https://plotly.com/r/static-image-export/>), which gives the possibility to save the images in the console also in .SVG format.

#### Tab 6: Download scores tables

The in the 6^th^ tab permits to download the results. The predictors table include all the metabolic scores that MiMIR could calculate in the uploaded dataset. Instead, the calibrated surrogate table will be available only if the phenotypic file was uploaded and includes only the value of the surrogate clinical variables calibrated using Platt scaling. It is possible to download these tables in .csv or .tsv based on the user’s preference.

#### Download the Analysis report

Finally, it will be also possible to download an R-Markdown file containing a report with all the details of the analysis that was just carried on. There are two possible files to download: “Download Analysis Report with accuracy analyses” will download the report containing also the results of the accuracy and calibration and will be available only if the phenotypic file was uploaded; otherwise, it will always be possible to download the report without the phenotypic information using: “Download Analysis Report without accuracy analyses”.

# Supplementary document 2 - Application to the Leiden Longevity study

## Study Description

The Leiden Longevity Study (LLS) consists of 421 long-lived families of European descent. Families were included if at least two long-lived siblings were alive and fulfilled the age criterion of 89 years or older for males and 91 years or older for females, representing <0·5% of the Dutch population in 2001 [8]. In total, 944 long-lived proband siblings (mean age = 94 years, range = 89-104), 1671 offspring (mean age = 61 years, range = 39-81) and 744 spouses thereof (mean age = 60 years, range = 36-79) were included. Registry-based follow-up until the 27^th^ of October 2016 was available for all participants. Metabolites were successfully quantified in 843 nonagenarians [LLS_SIBS], 1157 of their offspring and 684 controls (LLS_PAROFF) using non-fasted EDTA plasma samples.

## Acknowledgements

The LLS has received funding from the European Union's Seventh Framework Programme (FP7/2007-2011) under grant agreement n° 259679. This study was supported by a grant from the Innovation-Oriented Research Program on Genomics (SenterNovem IGE05007), the Centre for Medical Systems Biology, and the Netherlands Consortium for Healthy Ageing (grants 05040202 and 050-060-810), all in the framework of the Netherlands Genomics Initiative, Netherlands Organization for Scientific Research (NWO), Unilever Colworth, and by BBMRI-NL, a Research Infrastructure financed by the Dutch government (NWO 184.021.007).

## Ethics statement

The Leiden Longevity Study protocol was approved by the Medical Ethical Committee of the Leiden University Medical Center before the start of the study (P01.113). In accordance with the Declaration of Helsinki, the Leiden Longevity Study obtained informed consent from all participants prior to their entering the study.

## Metabolic features


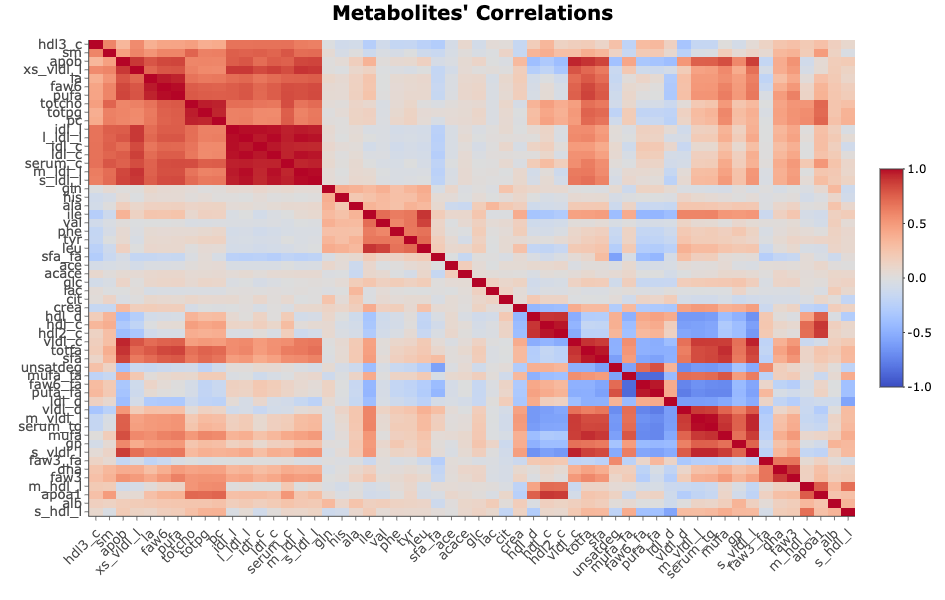


| **[B]**  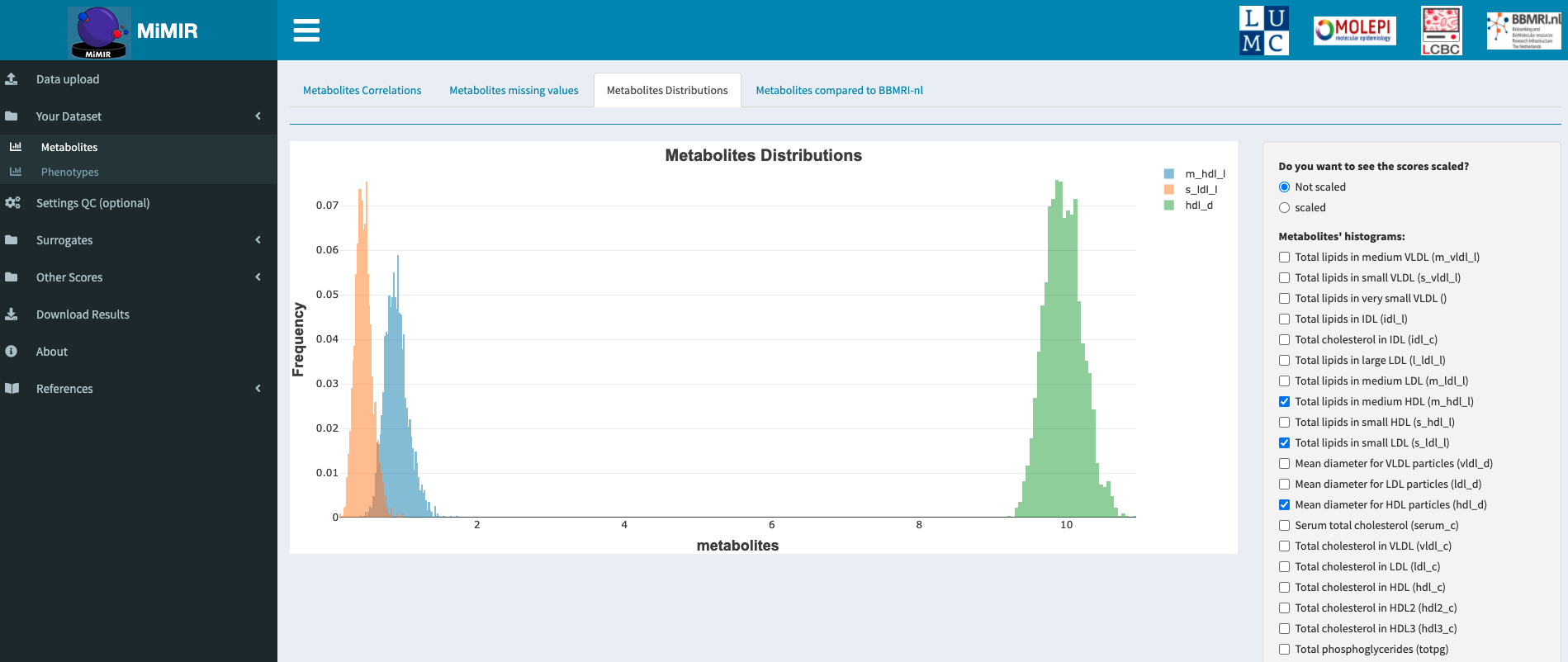 | **[C]**  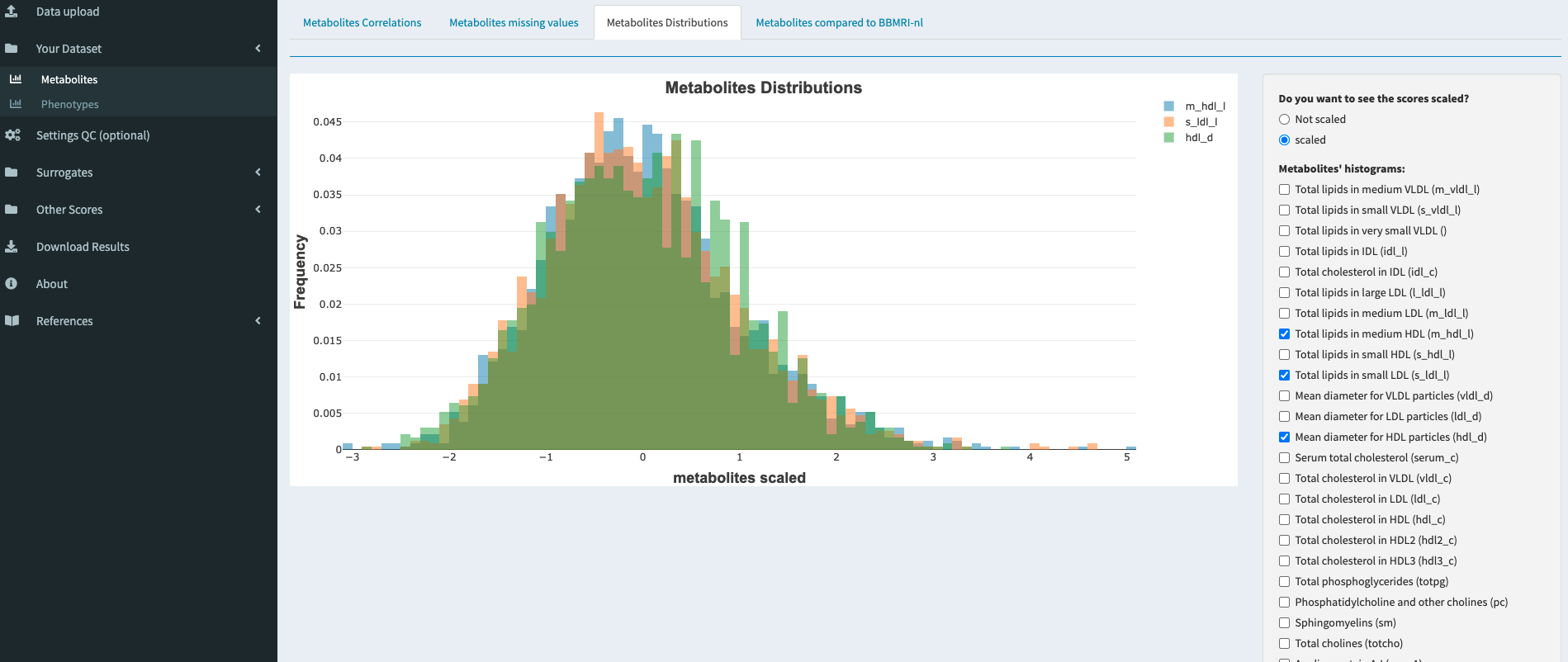 |
| --- | --- |

**Figure S31:** Metabolic features in LLS-PAROFFs. [A] Correlation of the metabolic features used in MetaboAge and the surrogate models, [B] Metabolites distributions of m_hdl_l, s_ldl_l and hdl_d and [C] their scaled version.

| [A]  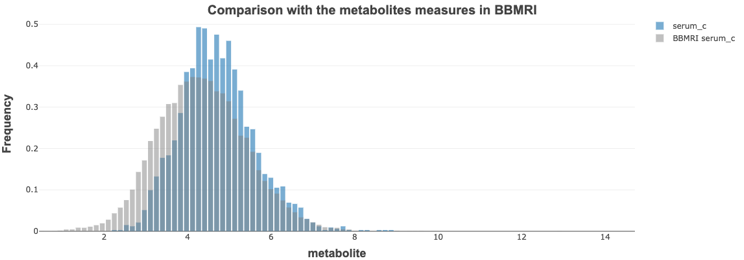 | [B]  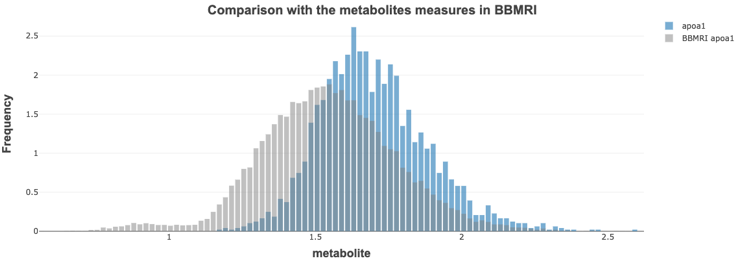 |
| --- | --- |
| [C]  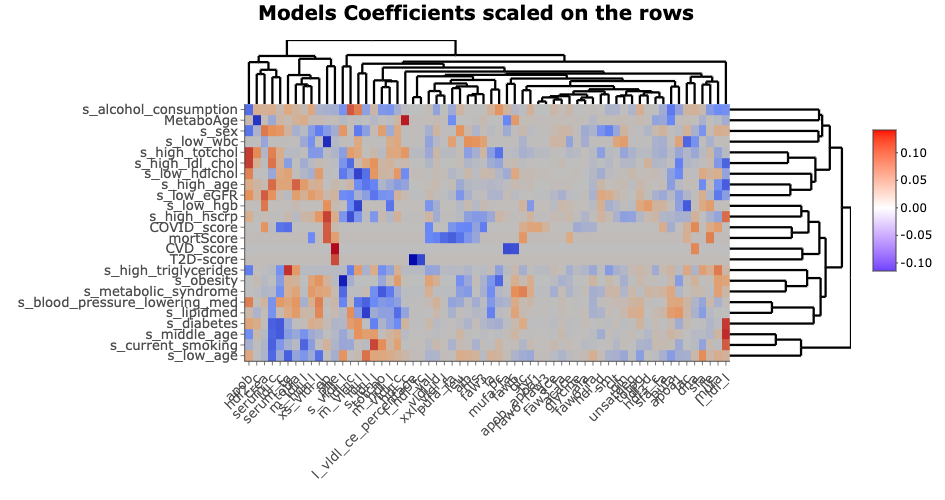 | |

**Figure S32:** Distribution of distribution of [A] serum_c and [B] apoa1 in LLS-PAROFF compared to the distribution in BBMRI-nl. [C] the relative importance of the metabolic features in each model. For instance, we can see that serum_c and apoa1 has a big impact surrogate ‘lipid medication’.

## Metabolic surrogates


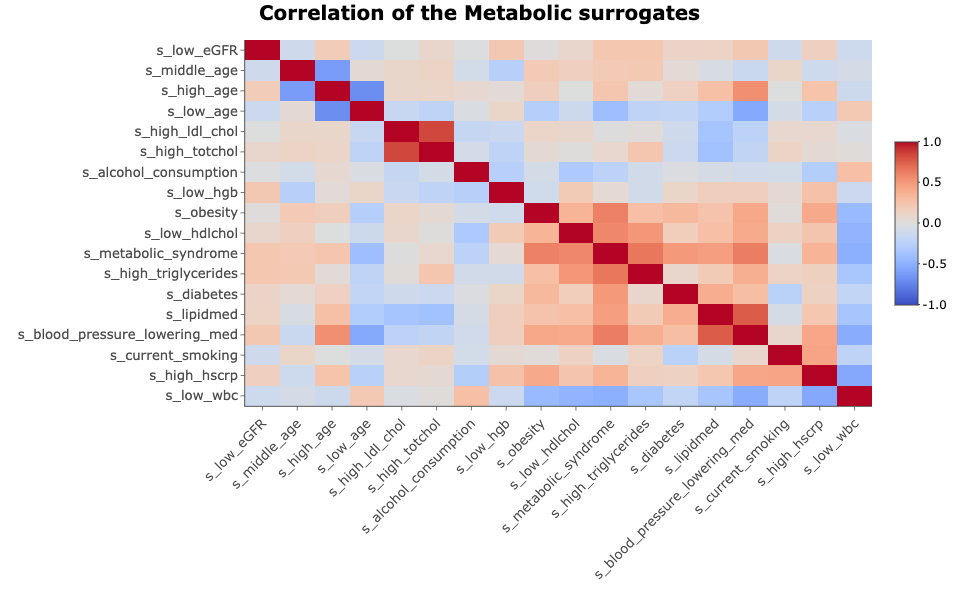


**Figure S33:** Pearson correlation between the surrogate variables. As shown in the original paper [3], the surrogate variables show even some high correlation, but they seem to be drive by the nature of the clinical variables included, not only by the fact that they are scores made with only 56 variables


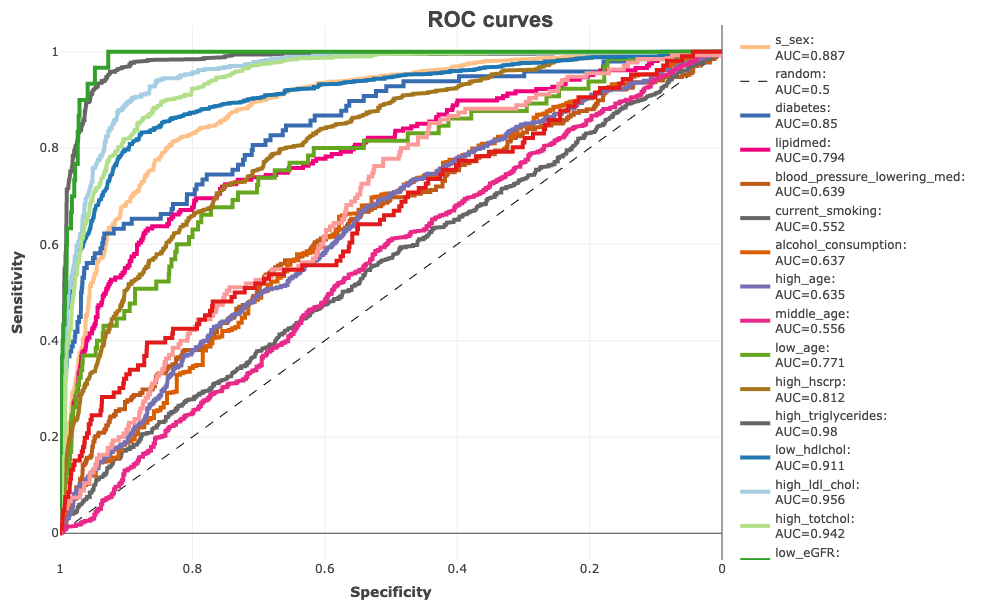


**Figure S34:** ROC curves for all the available metabolic surrogates.


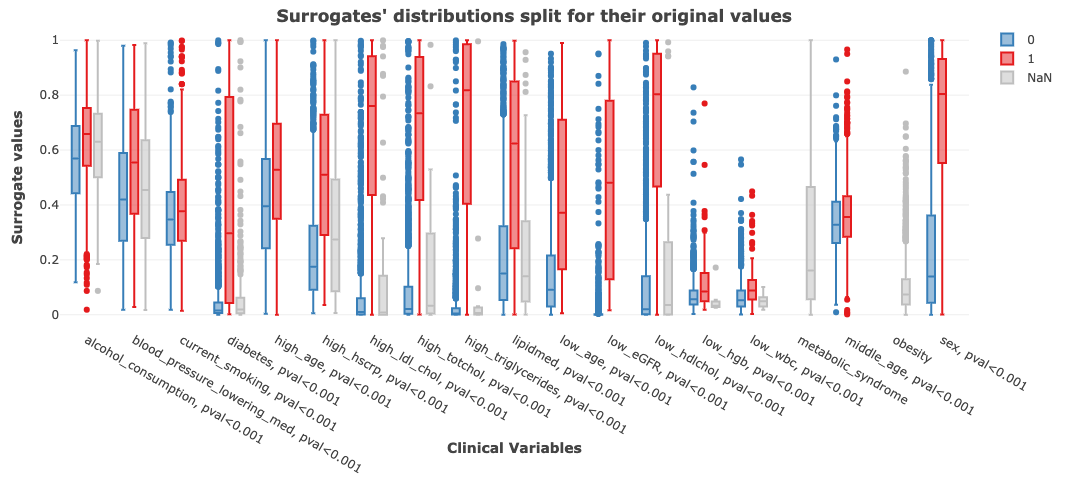


**Figure S35:** T-test indicating if the surrogate variables could split well the original clinical variables in LLS-PO. Generally, all the t-tests indicate that there is a good separation between TRUE/FALSE, however some variables have a clearer separation (e.g. ‘sex’) than others (e.g. ‘alcohol consumption’). Moreover, it is possible to visualize where the missing variables lie in the distributions of the surrogates. For example, we can notice how the missing values in the diabetes status generally indicates lower chance of diabetes.


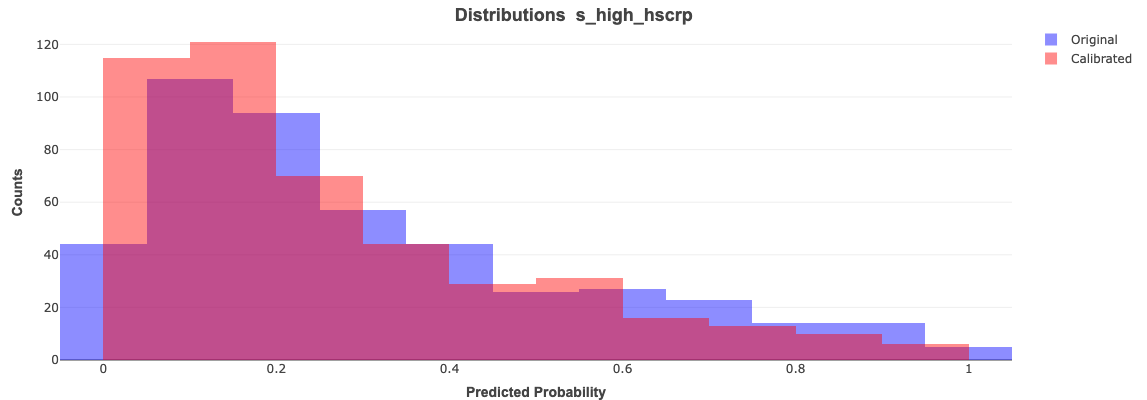


**Figure S36:** Distribution of the surrogate ‘*high hscrp*’ (blue) and its calibrated version (red) split in 10 bins.

| [A]  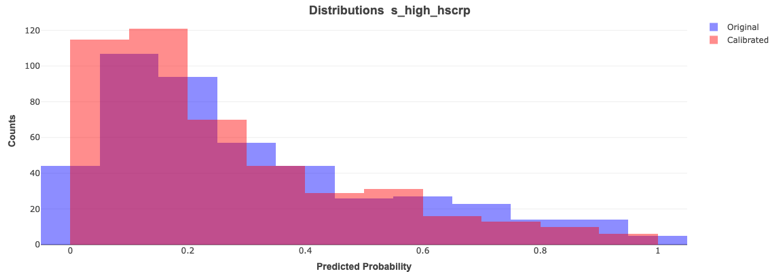 | [B]  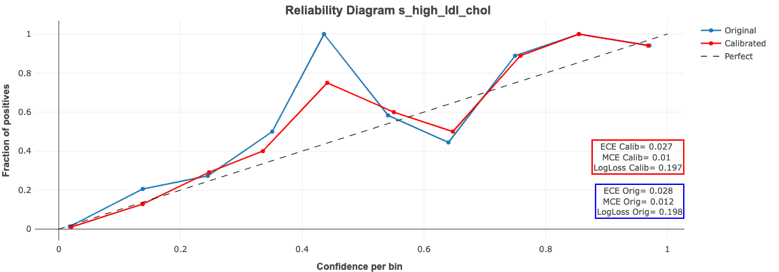 |
| --- | --- |

**Figure S37:** [A] Distribution split in 10 bins and [B] reliability histogram for ‘*high ldl chol*’ (blue) and its calibrated version (red) split in 10 bins. We can see that ${\Delta ECE}_{(calib-orig)}= -0.01$, ${\Delta MCE}_{(calib-orig)}= -0.001$, ${LogLoss}_{(calib-orig)}= -0.001$.

## Metabolic scores


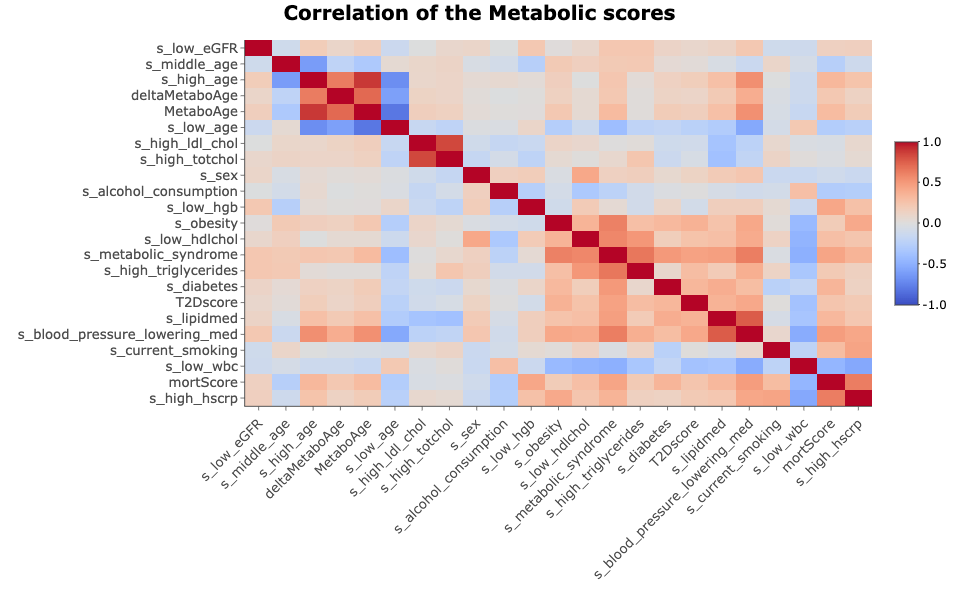


**Figure S38** Correlation of all the metabolomics-based scores. We can notice how some scores have high correlations, for instance, between the mortality score and the surrogate ‘*high hscrp*’.


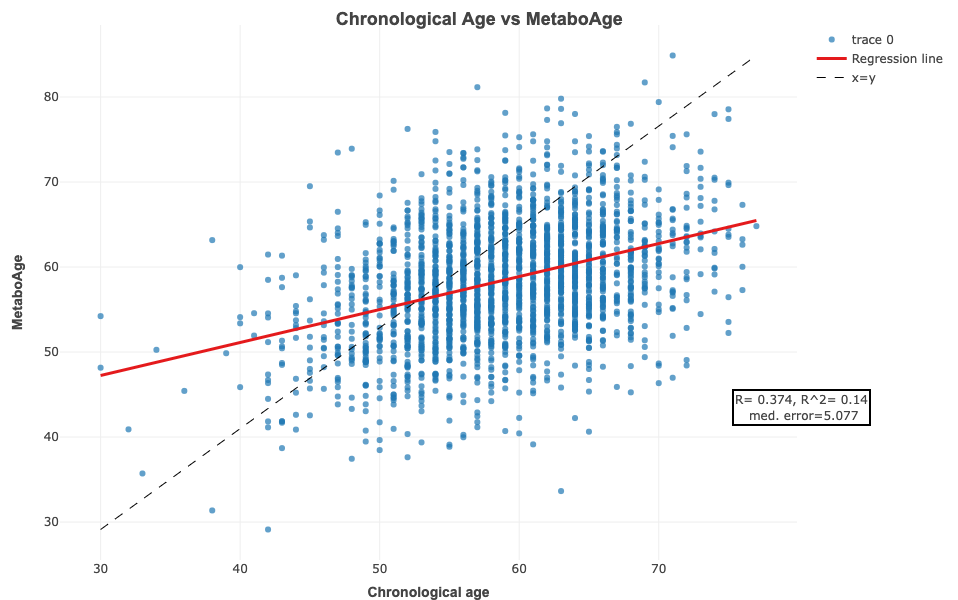


**Figure S39:** Scatterplot comparing chronological age to MetaboAge. The correlation between the two measures is around 0.374 and the median error 5.07 years.


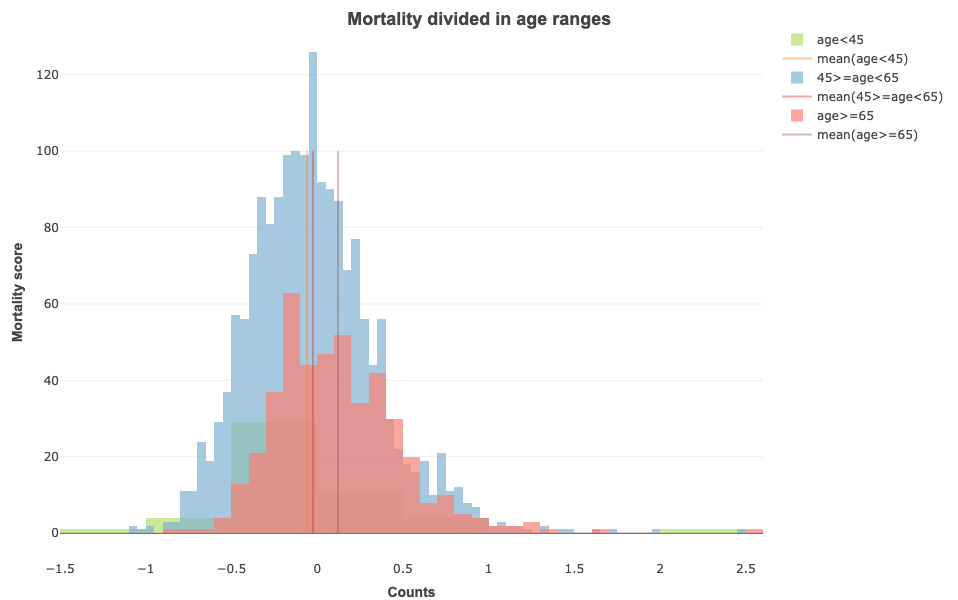


**Figure S40:** The distribution of the mortality score divided in three age ranges: lower than 45 years old (green), between 45 and 65 years old (blue) and higher than 65 years old (red). We can see how the higher the age is the higher the median of the mortality scores increases.


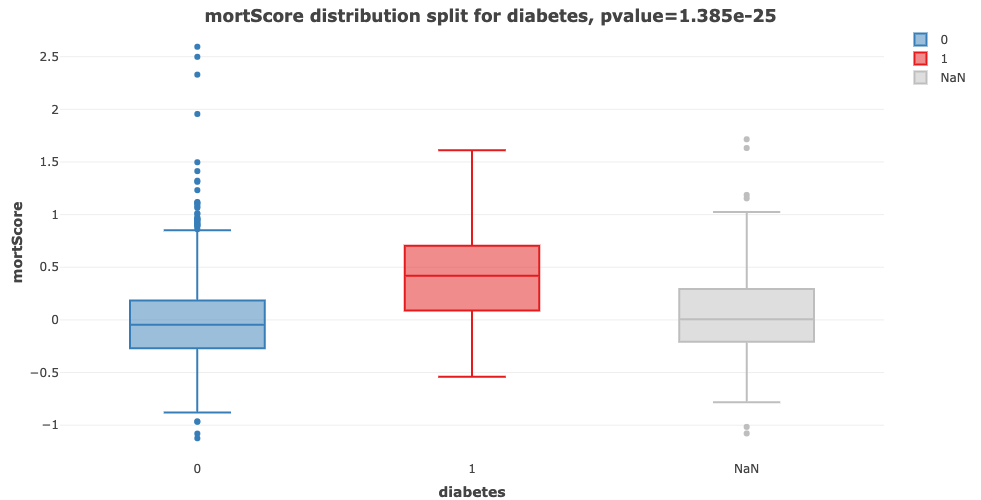


**Figure S41:** Comparison of the mortality score between diabetics and non-diabetics. The diabetics people have an increased mortality score with a highly significant difference (t-test p-value= 1.4 ${10}^{-25}$).


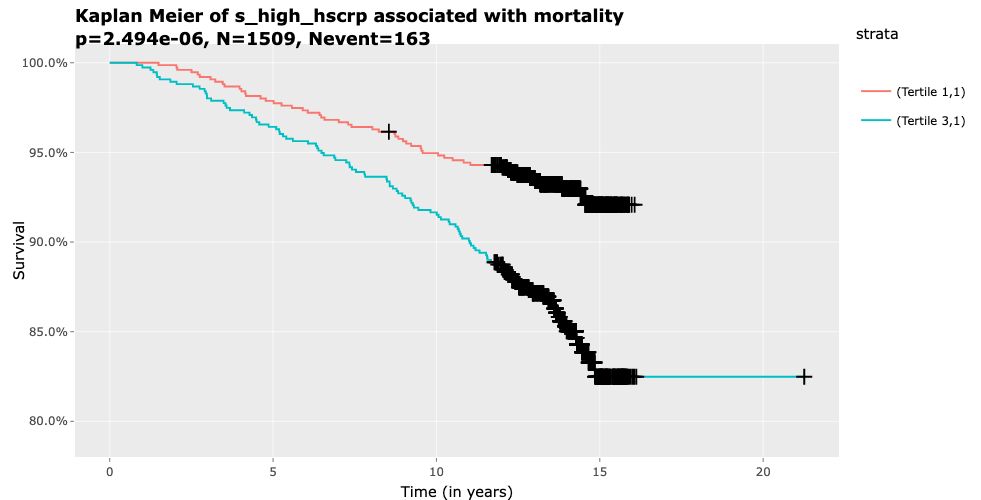


**Figure S42:** Kaplan Meier comparing the Time to Death of the participants divided according to the ‘*high hscrp*’, where the first tertiles includes participants with a lower ‘*high hscrp*’, while third tertiles the ones with a higher ‘*high hscrp*’. We can notice how people with a higher level ‘*high hscrp*’ are more inclined to die faster.


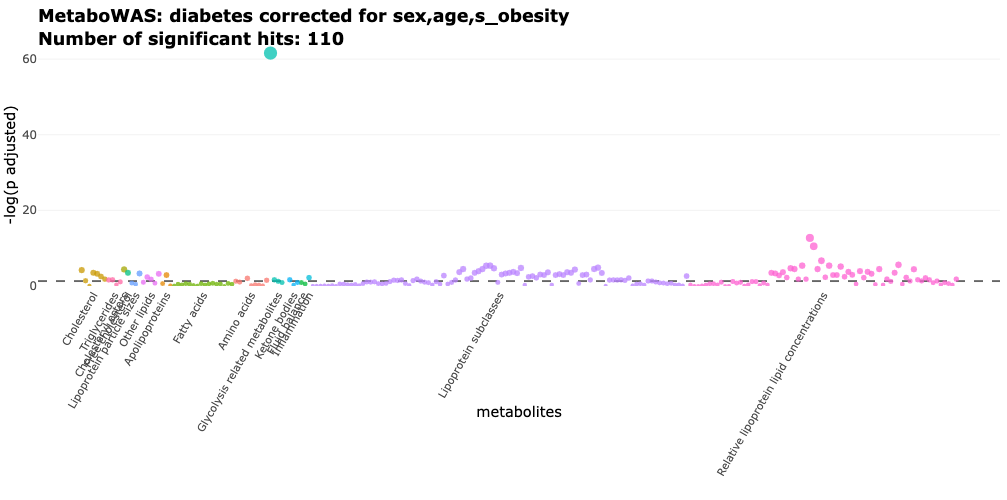


**Figure S43:** Results of a MetaboWAS for diabetes corrected for age, sex and surrogate ‘*obesity*’. Not surprisingly, the most significantly associated metabolite is glucose


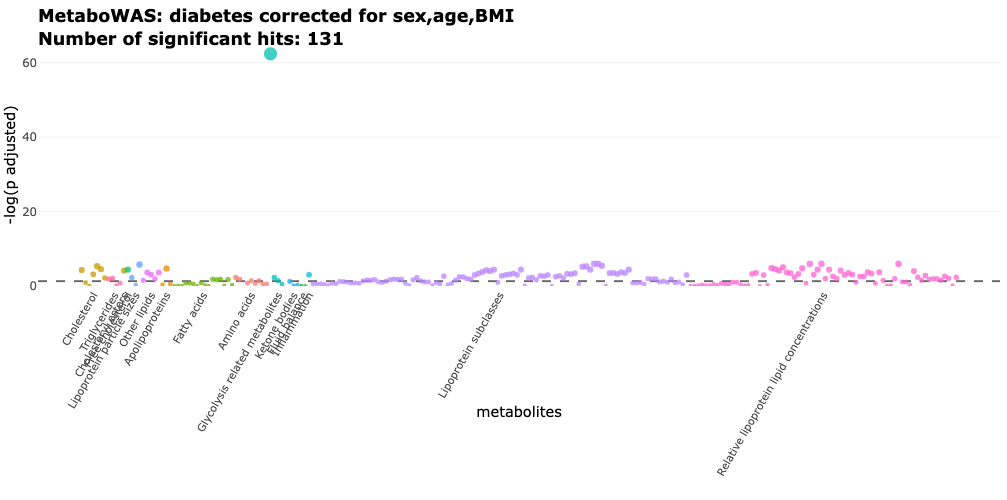


**Figure S44:** Results of a MetaboWAS for diabetes corrected for age, sex and BMI. Not surprisingly, the most significantly associated metabolite is still glucose.

# Supplementary document 3 – The synthetic dataset

MiMIR is shipped with an example data which was created as a synthetic dataset using the package “synthpop” [9]. Synthpop draws the synthetic values from the distributions of each variable in a reference study, while preserving the statistical properties and relationships with the other variables. In this way the dataset should resemble the reference study, as it preserves correlations between variables. The synthpop package was used to simulate 500 samples whose metabolite levels and phenotype data were modeled after the Partner-Offspring generation (LLS-PO) of the Leiden Longevity Study (median age = 59 years) [8]. The synthpop package mimics also the missingness of the original dataset. Hence, if a variable is completely missing in the original data, that will also be missing also in the synthetic dataset. As metabolic syndrome and obesity phenotypes were not available in LLS-PO, they are also missing in the synthetic dataset. For a detailed explanation of how the synthpop package works we refer to the paper by Nowok et al. [9]. The synthpop package allows also to draw useful plots to compare the distributions of the measured and the synthetic variables. Therefore, to show that the synthetic dataset is a faithful representation of samples in LLS_PO, we decided to include also these plots.

|  |  |
| --- | --- |
|  |  |
|  |  |

**Figure S44:** Comparing the phenotypic variables observed in LLS-PAROFFs (dark blue) with the ones created with synthpop (light blue).

|  |  |
| --- | --- |
|  |  |
|  |  |
|  |  |
|  |  |
|  |  |
|  |  |
|  |  |
|  |  |

**Figure S44:** Comparison of the metabolic features observed in LLS-PAROFFs (dark blue) with the ones created with synthpop (light blue).

# References

[1] J. Deelen *et al.*, ‘A metabolic profile of all-cause mortality risk identified in an observational study of 44,168 individuals’, *Nat. Commun.*, vol. 10, no. 1, pp. 1–8, Aug. 2019, doi: 10.1038/s41467-019-11311-9.

[2] van den Akker Erik B. *et al.*, ‘Metabolic Age Based on the BBMRI-NL 1H-NMR Metabolomics Repository as Biomarker of Age-related Disease’, *Circ. Genomic Precis. Med.*, vol. 13, no. 5, pp. 541–547, Oct. 2020, doi: 10.1161/CIRCGEN.119.002610.

[3] D. Bizzarri, M. J. T. Reinders, M. Beekman, P. E. Slagboom, Bbmri-nl, and E. B. van den Akker, ‘1H-NMR metabolomics-based surrogates to impute common clinical risk factors and endpoints’, *EBioMedicine*, vol. 75, p. 103764, Jan. 2022, doi: 10.1016/j.ebiom.2021.103764.

[4] Nightingale Health UK Biobank Initiative, H. Julkunen, A. Cichońska, P. E. Slagboom, and P. Würtz, ‘Metabolic biomarker profiling for identification of susceptibility to severe pneumonia and COVID-19 in the general population’, *eLife*, vol. 10, p. e63033, May 2021, doi: 10.7554/eLife.63033.

[5] A. V. Ahola-Olli *et al.*, ‘Circulating metabolites and the risk of type 2 diabetes: a prospective study of 11,896 young adults from four Finnish cohorts’, *Diabetologia*, vol. 62, no. 12, pp. 2298–2309, 2019, doi: 10.1007/s00125-019-05001-w.

[6] P. Würtz *et al.*, ‘Metabolite profiling and cardiovascular event risk: a prospective study of 3 population-based cohorts’, *Circulation*, vol. 131, no. 9, pp. 774–785, Mar. 2015, doi: 10.1161/CIRCULATIONAHA.114.013116.

[7] ‘Plotly R Graphing Library’. https://plotly.com/r/ (accessed Jul. 29, 2021).

[8] M. Schoenmaker *et al.*, ‘Evidence of genetic enrichment for exceptional survival using a family approach: the Leiden Longevity Study’, *Eur. J. Hum. Genet.*, vol. 14, no. 1, Art. no. 1, Jan. 2006, doi: 10.1038/sj.ejhg.5201508.

[9] B. Nowok, G. M. Raab, and C. Dibben, ‘synthpop: Bespoke Creation of Synthetic Data in R’, *J. Stat. Softw.*, vol. 74, no. 1, Art. no. 1, Oct. 2016, doi: 10.18637/jss.v074.i11.

[10] *NightingaleHealth/ggforestplot*. Nightingale Health, 2021. Accessed: Jul. 27, 2021. [Online]. Available: https://github.com/NightingaleHealth/ggforestplot

[11] W. T. Friedewald, R. I. Levy, and D. S. Fredrickson, ‘Estimation of the Concentration of Low-Density Lipoprotein Cholesterol in Plasma, Without Use of the Preparative Ultracentrifuge’, *Clin. Chem.*, vol. 18, no. 6, pp. 499–502, Jun. 1972, doi: 10.1093/clinchem/18.6.499.

[12] Carrero Juan Jesus, Andersson Franko Mikael, Obergfell Achim, Gabrielsen Anders, and Jernberg Tomas, ‘hsCRP Level and the Risk of Death or Recurrent Cardiovascular Events in Patients With Myocardial Infarction: a Healthcare‐Based Study’, *J. Am. Heart Assoc.*, vol. 8, no. 11, p. e012638, Jun. 2019, doi: 10.1161/JAHA.119.012638.

[13] A. J. Flint *et al.*, ‘Body mass index, waist circumference, and risk of coronary heart disease: a prospective study among men and women’, *Obes. Res. Clin. Pract.*, vol. 4, no. 3, pp. e171–e181, 2010, doi: 10.1016/j.orcp.2010.01.001.

[14] L. Dean and L. Dean, *Blood Groups and Red Cell Antigens*. National Center for Biotechnology Information (US), 2005.

[15] D. Bizzarri, M. J. T. Reinders, M. Beekman, P. E. Slagboom, BBMRI-NL, and E. B. van den Akker, ‘1H-NMR metabolomics-based surrogates to impute common clinical risk factors and endpoints’, *medRxiv*, p. 2021.07.19.21258470, Jul. 2021, doi: 10.1101/2021.07.19.21258470.

[16] E. Crimmins, S. Vasunilashorn, J. K. Kim, and D. Alley, ‘BIOMARKERS RELATED TO AGING IN HUMAN POPULATIONS’, *Adv. Clin. Chem.*, vol. 46, pp. 161–216, 2008.

[17] S. A. Williams *et al.*, ‘Plasma protein patterns as comprehensive indicators of health’, *Nat. Med.*, vol. 25, no. 12, pp. 1851–1857, Dec. 2019, doi: 10.1038/s41591-019-0665-2.

[18] P. Würtz *et al.*, ‘Metabolic Signatures of Adiposity in Young Adults: Mendelian Randomization Analysis and Effects of Weight Change’, *PLoS Med.*, vol. 11, no. 12, Dec. 2014, doi: 10.1371/journal.pmed.1001765.

[19] J. C. Platt, ‘Probabilistic Outputs for Support Vector Machines and Comparisons to Regularized Likelihood Methods’, in *Advances in Large Margin Classifiers*, 1999, pp. 61–74.

[20] M. E. Maros *et al.*, ‘Machine learning workflows to estimate class probabilities for precision cancer diagnostics on DNA methylation microarray data’, *Nat. Protoc.*, vol. 15, no. 2, Art. no. 2, Feb. 2020, doi: 10.1038/s41596-019-0251-6.

[21] M. H. DeGroot and S. E. Fienberg, ‘The Comparison and Evaluation of Forecasters’, *J. R. Stat. Soc. Ser. Stat.*, vol. 32, no. 1/2, pp. 12–22, 1983, doi: 10.2307/2987588.

[22] A. Niculescu-Mizil and R. Caruana, ‘Predicting good probabilities with supervised learning’, in *Proceedings of the 22nd international conference on Machine learning - ICML ’05*, Bonn, Germany, 2005, pp. 625–632. doi: 10.1145/1102351.1102430.

[23] C. Guo, G. Pleiss, Y. Sun, and K. Q. Weinberger, ‘On Calibration of Modern Neural Networks’, *ArXiv170604599 Cs*, Aug. 2017, Accessed: Jun. 14, 2021. [Online]. Available: http://arxiv.org/abs/1706.04599

[24] J. Nixon, M. W. Dusenberry, L. Zhang, G. Jerfel, and D. Tran, ‘Measuring Calibration in Deep Learning’, p. 4.

[25] A. G. Barnett, J. C. van der Pols, and A. J. Dobson, ‘Regression to the mean: what it is and how to deal with it’, *Int. J. Epidemiol.*, vol. 34, no. 1, pp. 215–220, Feb. 2005, doi: 10.1093/ije/dyh299.

[26] D. Ashby, ‘Practical statistics for medical research. Douglas G. Altman, Chapman and Hall, London, 1991. No. of pages: 611. Price: £32.00’, *Stat. Med.*, vol. 10, no. 10, pp. 1635–1636, 1991, doi: 10.1002/sim.4780101015.
